# Spectral principal axis system (SPAS) and tuning of tensor-valued encoding for microscopic anisotropy and time-dependent diffusion in the rat brain

**DOI:** 10.1162/IMAG.a.35

**Published:** 2025-06-11

**Authors:** Samo Lasič, Nathalie Just, Markus Nilsson, Filip Szczepankiewicz, Matthew Budde, Henrik Lundell

**Affiliations:** Danish Research Centre for Magnetic Resonance, Department of Radiology and Nuclear Medicine, Copenhagen University Hospital—Amager and Hvidovre, Copenhagen, Denmark; Department of Diagnostic Radiology, Lund University, Lund, Sweden; Department of Clinical Sciences Lund, Radiology, Lund University, Lund, Sweden; Medical Radiation Physics, Clinical Sciences Lund, Lund University, Lund, Sweden; Department of Neurosurgery, Medical College of Wisconsin, Milwaukee, WI, United States; Magnetic Resonance Section, DTU Health Tech, Technical University of Denmark, Kgs. Lyngby, Denmark

**Keywords:** diffusion MRI, tensor-valued encoding, power spectrum, time-dependent diffusion, restricted diffusion, gradient waveform

## Abstract

Tensor-valued encoding in diffusion MRI allows probing of microscopic anisotropy in tissue, however, time-dependent diffusion (TDD) can bias results unless b-tensors are carefully tuned to account for TDD. We propose two novel strategies for tuning b-tensors to enable accurate measurements without interference from TDD due to restricted diffusion. The first strategy involves identifying encoding tensor projections that yield equal mean diffusivities (MD), providing robust tuning across a wide range of diffusion spectra. The second strategy uses geometric averaging of signals, ensuring tuning regardless of the diffusion spectra. Importantly, the same encoding waveforms used for geometric averaging to probe microscopic anisotropy (µA) can also generate an independent contrast due to TDD. This is enabled by considering spectral anisotropy of encoding and defining the spectral principal axis system (SPAS), which unfolds TDD as an additional independent dimension in tensor-valued encoding. Projections of encoding waveforms along the SPAS axes allow for the simultaneous acquisition of independent contrasts due to both µA and TDD within a single multidimensional diffusion encoding protocol. Additionally, SPAS projections inherit useful properties from the reference tensor, such as optimized b-value, motion nulling, and minimal concomitant field effects. This framework is demonstrated through simulations of various restricted diffusion compartments. Experimental validation on perfusion-fixed and*in vivo*rat brains highlights the method’s potential for enhanced microstructural specificity. In addition to mapping MD, fractional anisotropy, and unbiased microscopic fractional anisotropy, we propose a model-free approach to independently map µA and TDD. This approach uses a minimal yet highly specific protocol, enabling the identification of distinct µA-TDD contrasts across different brain regions, including details in cortical gray matter, choroid plexus, dentate gyrus of the hippocampus, and white matter.

## 1 Introduction

Diffusion magnetic resonance imaging (dMRI) is commonly used as a non-invasive probe of brain microstructure (Tournier et al., 2011). The technique is very sensitive to changes in microstructure, but its specificity is limited because the dMRI signal reflects a mix of tissue characteristics, including cell shape, size, orientation, and permeability. This problem is exacerbated when the tissue is heterogeneous, that is, when the relatively large imaging voxel contains a mixture of tissues with different microstructural features (Jeurissen et al., 2013;Mulkern et al., 2000). One approach to increase specificity is to employ increasingly more sophisticated biophysical models (Novikov, Kiselev, et al., 2018;Olesen et al., 2022;Palombo et al., 2020), which often rely on fragile assumptions (Jelescu et al., 2020;Jones et al., 2013;Lampinen et al., 2023). Another approach is to use encoding strategies that aim to increase specificity at the acquisition stage (Topgaard, 2020). The goal is to disentangle different confounding factors, such as molecular exchange (Callaghan & Furó, 2004;Chakwizira, Westin, et al., 2023;Lasič et al., 2011), restricted and hindered diffusion (Balinov et al., 1993;Lasič et al., 2006,^2009^;Neuman, 1974;Stepišnik, 1993), incoherent flow (Ahlgren et al., 2016), microscopic and macroscopic anisotropy (Jespersen et al., 2013;Lasič et al., 2014;Nilsson et al., 2020;Szczepankiewicz et al., 2016;Topgaard, 2017,^2019^;Westin et al., 2014,^2016^), and relaxation (de Almeida Martins et al., 2020;De Almeida Martins & Topgaard, 2018;Lampinen, Szczepankiewicz, et al., 2020;Veraart et al., 2018).

Traditional diffusion encoding employs pulse field gradients (PFG) (Stejskal, 1965;Stejskal & Tanner, 1965) to sensitize the signal to diffusion along a single direction. Such encoding is used in diffusion tensor imaging (DTI) (Basser et al., 1994) and diffusion kurtosis imaging (DKI) (Jensen et al., 2005). However, these approaches entangle information about tissue anisotropy on microscopic and macroscopic scales. In tensor-valued encoding (Jespersen et al., 2013,^2014^;Lasič et al., 2014;Nilsson et al., 2020;Szczepankiewicz et al., 2016;Topgaard, 2017,^2019^;Westin et al., 2016), gradients are played out simultaneously but asynchronously along multiple orthogonal directions to yield b-tensors of varying shapes, which enables microstructure assessment unconfounded by the orientation distribution of anisotropic microstructures and morphological heterogeneity within macroscopic imaging voxels. Tensor-valued encoding can probe microscopic fractional anisotropy (µFA), for example, by using a combination of linear tensor encoding (LTE) and planar tensor encoding (PTE) (Jespersen et al., 2013) or with LTE and spherical tensor encoding (STE) (Lasič et al., 2014;Szczepankiewicz et al., 2016). Adopting more general approaches to tensor-valued encoding, not limited to the PFG paradigm, opens up venues for optimizations in terms of b-tensor shapes used for different hardware and physiological constraints (Szczepankiewicz, Westin, et al., 2021) and encoding waveforms suitable to maximize encoding strength (Sjölund et al., 2015), minimize effects of concomitant fields (Szczepankiewicz, Westin, et al., 2019), background gradients (Szczepankiewicz & Sjölund, 2021), or enable varying degree of motion compensation (Lasič et al., 2020;Szczepankiewicz, Teh, et al., 2021).

Analysis of tensor-valued encoding data often assumes multi-Gaussian diffusion. This means that the measured signals depend only on the b-tensor size and shape but not on the temporal characteristics of the diffusion encoding gradient waveforms. However, restricted diffusion in tissue could cause time-dependent diffusion (TDD), where the signals and the associated apparent diffusivities depend on the encoding waveform time scales. Different b-tensors may utilize encoding waveforms with different time scales and thereby feature different sensitivities to TDD (Lasič et al., 2022;Lundell et al., 2018,^2020^;Lundell, Nilsson, Dyrby, et al., 2019;Lundell & Lasič, 2020;Teh et al., 2021). Combining b-tensors with different TDD sensitivities could significantly bias estimation of microscopic anisotropy (µA) or the separation of isotropic and anisotropic sources of the mean kurtosis. Such conditions may be prominent in preclinical settings using shorter encoding times (Ianuş et al., 2018;Lundell, Nilsson, Dyrby, et al., 2019) or in tissues with larger cells like in the heart (Lundell et al., 2020;Teh et al., 2021). For unbiased assessment of µA, b-tensors need to be*tuned*for equal TDD sensitivity to yield equal mean diffusivities (MD) (Lundell et al., 2020;Lundell, Nilsson, Dyrby, et al., 2019;Lundell & Lasič, 2020).

This work considers TDD due to restricted diffusion in tensor-valued diffusion encoding with two main objectives: (1) to reduce bias in µA assessment and (2) to obtain two independent contrasts due to µA and TDD using a minimal set of diffusion encoding waveforms. We propose two approaches for tuning LTE to an arbitrary b-tensor shape.

The first approach uses a shorter protocol with a single LTE waveform, ideal when the primary goal is to assess µA unconfounded by TDD. The tuned LTE waveform is selected as the one-dimensional projection of an STE waveform that yields the TDD profile most similar to that of the STE. This approach provides tuning relative to the sample, thus requiring prior knowledge of the expected range of restriction sizes. The second approach involves a longer protocol with multiple LTE waveforms, using geometric averaging to obtain the tuned LTE data. This method achieves tuning that is independent of restricted diffusion effects in the tissue. In addition, we aim to maximize the experimental yield, by allowing acquisition of independent contrasts sensitive to µA and TDD within a single multidimensional protocol. This is achieved by defining the spectral principal axis system (SPAS) of encoding (Lasič et al., 2022) and combining it with the geometric averaging approach. The effectiveness of these methods is demonstrated through simulations and experiments on both perfusion-fixed and*in vivo*rat brains.

## 2 Theory

In this section, we present a framework for reducing the confounding effects of time-dependent diffusion (TDD) in tensor-valued encoding for microscopic anisotropy (µA) assessment. We also introduce the spectral principal axis system (SPAS) to probe both µA and TDD using the same set of encoding waveforms.

We begin by revisiting the principles of tensor-valued encoding, highlighting the need to account for TDD due to restricted diffusion. Using frequency-domain analysis under the Gaussian phase approximation (GPA), we identify two critical encoding attributes:*spectral trace*and*spectral anisotropy*. Spectral trace is the key parameter for tuning b-tensors, while spectral anisotropy reflects how sensitivity to TDD varies with encoding orientation. These concepts underpin the SPAS, which defines an orthogonal set of projections optimized for distinct TDD sensitivities.

We then outline two approaches for tuning: one based on matching apparent mean diffusivity (MD) and another using geometric signal averaging. Both strategies help mitigate the confounding effect of TDD in µA assessment, with geometric averaging offering more robust tuning and enabling simultaneous probing of µA and TDD within a single encoding protocol.

### 2.1 Ensemble average signal in tensor-valued encoding

When imaging voxels contain multiple compartments with different diffusivities, anisotropies, and orientations, the signal attenuation is generally multiexponential. At low b-values, the signal attenuation can be approximated by a cumulant expansion

$$\log[\langle S\rangle ]=\log[\langle S_{0}\rangle ]-b\text{MD}+\frac{b^{2}}{2}V_{D}-\cdots ,$$ (1)

where MD is the apparent mean diffusivity and$V_{D}$is the apparent diffusion variance, related to the “mean kurtosis” (MK) by$V_{D}=\frac{1}{3}{\text{MD}}^{2}\text{MK}$(Szczepankiewicz et al., 2016;Westin et al., 2016). Note that MD and$V_{D}$are rotationally invariant measures derived from the powder-averaged signal. In tensor-valued encoding, the anisotropy or shape of the b-tensors provides an independent encoding dimension, allowing us to separate$V_{D}$into isotopic ($V_{\text{I}}$) and anisotropic ($V_{\text{A}}$) contributions,$V_{D}=V_{\text{I}}+V_{\text{A}}.$For axially symmetric encoding, we have

$$\log[\langle S\rangle ]\approx \log[\langle S_{0}\rangle ]-b\text{MD}\;+\;\frac{b^{2}}{2}[V_{\text{I}}+b_{\Delta }^{2}V_{\text{A}}],$$ (2)

where$b_{\Delta }^{2}$is 0 for spherical (STE) and 1 for linear (LTE) tensor encoding (Eriksson et al., 2015;Topgaard, 2017). By varying the b-tensor shape, we can probe$V_{\text{A}}$, assuming the encoding of MD and$V_{\text{I}}$remains independent of b-tensor shape, which holds when the diffusion process is approximately Gaussian.

Now, consider a combination of STE and LTE encodings, commonly used to assess microscopic anisotropy (Lasič et al., 2014;Nilsson et al., 2020;Szczepankiewicz et al., 2016;Topgaard, 2017,^2019^;Westin et al., 2016). The signal difference between STE and LTE at a given b-value is approximately

Δlog[〈S〉]≈−bΔMD + µA, (3)

where we define the microscopic anisotropy contrast as

$$\text{µA}\equiv \frac{b^{2}}{2}\Delta V_{D}=\underset{\Delta \text{MD}\to 0}{\lim}\Delta \log[\langle S\rangle ],$$ (4)

For Gaussian diffusion,ΔMD=0and$\Delta V_{D}$is proportional to the microscopic fractional anisotropy (µFA) (Jespersen et al., 2013;Lasič et al., 2014), defined as

$${\text{µFA}}^{2}=\frac{3}{2}{\left(1\;+\;\frac{2}{5}\frac{{\text{MD}}^{2}}{\Delta V_{D}}\right)}^{-1}.$$ (5)

In contrast to FA, µFA removes the effect of orientation dispersion, which is related to the order parameter (Lasič et al., 2014). Note that the definition of µFA above differs slightly from that inSzczepankiewicz et al. (2016), where the isotropic diffusion variance was added to${\text{MD}}^{2}$. Furthermore, µFA^2^maps, visualized in this work, scale similarly as the µA maps as a function of$\Delta V_{D}$.

Restricted diffusion may lead toΔMD≠0, introducing bias in the assessment of µA and µFA. For example, if LTE measures diffusion at longer time scales than STE, the apparent MD from LTE may be lower due to restricted diffusion, leading toΔMD<0.This could increase the signal separation inEq. (3), falsely suggesting higher µA and positively biasing µFA estimates.

An illustrative example of the potential bias introduced by combining “oscillating” STE gradients (Topgaard, 2013) with more efficient pulsed-like LTE gradients (Lasič et al., 2014) is provided by experiments in densely packed isotropic yeast cells (Lundell, Nilsson, Dyrby, et al., 2019). In these experiments, high signal differences could have been erroneously interpreted as high cell anisotropy if TDD effects were not considered. Such a combination of different gradient timings for LTE and STE is reminiscent of experiments employing oscillating gradients (Callaghan & Stepišnik, 1996;Gore et al., 2001;Stepišnik, 1993,^1998^,^1999^) or a combination of oscillating and pulsed gradients (X. Jiang et al., 2017;Reynaud et al., 2016a;Reynaud, 2017) designed to probe restriction sizes. Although combining waveforms with different sensitivities to TDD is useful for probing cell sizes, it is generally undesirable in tensor-valued encoding. This underscores the importance of carefully tuning b-tensors to minimize the confounding effects of TDD to ensure unbiased assessments of µA. The following sections will address this challenge by introducing a frequency-domain analysis that forms the basis for understanding and managing TDD effects in tensor-valued encoding.

### 2.2 Time-dependent diffusion with tensor-valued encoding—the frequency-domain analysis

The effects of TDD due to restricted diffusion can be analyzed in the frequency domain using the Gaussian phase approximation (GPA) (Ianuş et al., 2013;Stepišnik, 1993,^1998^,^1999^). This approach can also be extended to the asynchronous diffusion encoding waveforms characteristic of tensor-valued encoding (Lundell & Lasič, 2020). In the first-order approximation, TDD is examined by analyzing the*encoding*and*diffusion power spectra*. Unlike LTE, tensor-valued encoding employs asynchronous gradientsg(t)along orthogonal axes, which are essential for tensorial diffusion weighting. In frequency domain, the signal attenuation is given via coupling of the*dephasing*and*velocity cross-power spectral densities*. For brevity we call them*encoding*and the*diffusion power spectra*(Lundell & Lasič, 2020). With the dephasing vector

$$\text{q}\left(t\right)=\gamma \int_{0}^{t}\text{g}\left(t^{\prime }\right)\text{d}t^{\prime },$$ (6)

and its Fourier transform

$$\text{q}\left(\omega \right)=\gamma \int_{-\infty }^{\infty }\text{q}\left(t\right){\text{e}}^{i\omega t}\text{d}t,$$ (7)

the tensorial 3 x 3 (*i*x*j*) components of the encoding power spectrum are defined as

$$s_{ij}(\omega )=q_{i}(\omega )\;q_{j}(-\omega ).$$ (8)

The b-tensor components are given by

$$b_{ij}=\frac{1}{2\text{π}}\int_{-\infty }^{\infty }s_{ij}(\omega )\;\text{d}\omega$$ (9)

and the b-value by

$$b=\frac{1}{\text{π}}\int_{0}^{\infty }s(\omega )\;\text{d}\omega ,$$ (10)

where

$$s(\omega )=\sum_{i=1}^{3}s_{ii}(\omega )$$ (11)

is the encoding*spectral trace*(seeFig. 1A). This is the key encoding attribute for*tuning*different b-tensors. Sinces(ω)is real valued and symmetric around 0, we can use 0 as the lower frequency bound inEq. (10).

**Fig. 1.**
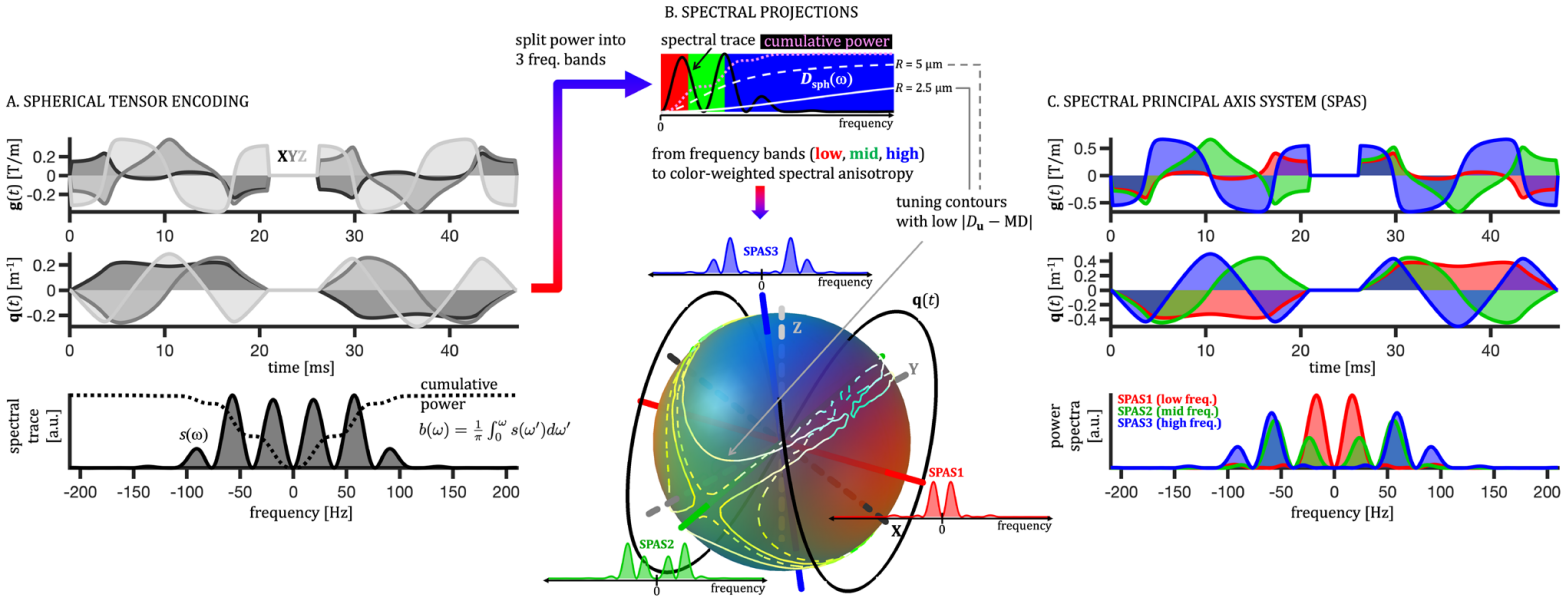
Spectral principal axis system (SPAS) and tuning contours for spherical tensor encoding (STE). (A) Effective gradient and dephasing waveforms (XYZ from darker to brighter gray), the spectral trace (shaded), and the cumulative encoding power (dashed line) for numerically optimized STE. Velocity compensation was achieved by changing the effective gradient polarity after the 180 RF pulse (Lundell & Lasič, 2020). (B) Color-coded projections of the STE cross-power spectral density and contours outlining 10% of the best tuned projections ($0.9<\frac{D_{\text{u}}}{\text{MD}}<1.1$) for spheres of two sizes (solid and dashed lines), where MD is the mean diffusivity (Eq. (15)) and$D_{\text{u}}$is the apparent diffusivity along a given projection (Eqs. (12)and(18)). The power spectrum is split into three frequency bands (RGB in the spectral band inset) based on the cumulative encoding power reaching 1/3 and 2/3 of the total power (b-value). The color coding of spectral anisotropy is based on the relative contributions from these three frequency bands (seeSection 2.4). The eigenvectors of the low-frequency filtered b-tensor provide the SPAS axes (SPAS1-3), shown in red, green, and blue along with the power spectra. Note that the XYZ axes generally do not coincide with the SPAS. (C) Linear tensor encoding (LTE) projections of STE along the SPAS axes (red, green, blue): effective gradient and dephasing waveforms and the corresponding encoding power spectra. The encoding power shifts from low to high frequencies for SPAS1 to SPAS3 corresponding to decreasing eigenvalues of the low-frequency filtered b-tensor. Note on color coding: RGB is used both to visualize spectral anisotropy (based on relative contributions from low-, mid-, and high-frequency bands) and to label the SPAS projections. This does not imply that SPAS axes isolate frequency bands. As seen in panel C, their spectra overlap, indicating that SPAS projections span all frequencies while emphasizing different TDD sensitivities.

The directional variation of the encoding power distribution is represented by spectral projections along a unit vector**u**as

$$s_{\text{u}}(\omega )=\sum_{i=1}^{3}\;\sum_{j=1}^{3}\;s_{ij}(\omega )\;u_{i}\;u_{j}.$$ (12)

This directional dependence of the encoding power distribution defines*spectral anisotropy*(SA), which reflects how sensitivity to TDD varies along different axes in tensor-valued encoding (Fig. 1). While the b-value (b-tensor trace) and b-tensor anisotropy suffice to characterize Gaussian diffusion, tuning (spectral trace) and spectral anisotropy add crucial encoding information to address TDD effects (Lundell & Lasič, 2020). These concepts are central to the encoding strategies discussed in the following sections.

Consider a single sub-ensemble of spins (tissue compartment) in the sense of an ergodic stochastic process of positions characterized by a common velocity autocorrelation function (Lundell & Lasič, 2020;Van Kampen, 2001). Such compartments could for example reside within intra- or extra-cellular spaces (Lasič et al., 2009;Novikov et al., 2011;Stepišnik, 1993) or represent incoherent flow (Kennan et al., 1994). With GPA (Ianuş et al., 2013;Stepišnik, 1993,^1998^,^1999^), the attenuation factor$\beta =-\ln(S\;/\;S_{0})$is obtained by integrating the inner product of the encoding and diffusion spectra over the entire frequency range,

$$\beta \approx \frac{1}{2\text{π}}\sum_{i=1}^{3}\;\sum_{j=1}^{3}\;\int_{-\infty }^{\infty }s_{ij}(\omega )\;D_{ij}(\omega )\text{ d}\omega .$$ (13)

Note that the symmetry of the encoding spectra,$s_{ij}(\omega )={\bar{s}}_{ji}(\omega )$, could be used to rewriteEq. (13)with 0 as the low integration limit (see equations 2.100–2.103 inLundell & Lasič, 2020). The diffusion spectrum from a single compartment can be expressed with rotation matrices**R**as

$$D_{ij}(\omega )=\sum_{k=1}^{3}R_{ki}\;R_{kj}\;\lambda_{k}(\omega ),$$ (14)

where$\lambda_{k}(\omega )$are diffusion eigenspectra (Lasič et al., 2009,^2022^;Stepišnik, 1993). This expression is useful to evaluate ensemble average signals (Lundell & Lasič, 2020) and was used in the simulations presented inFigure 4.

### 2.3 Two approaches for tuning b-tensors

In this section we elaborate on what we mean by tuning and suggest two approaches for tuning considering STE as the tuning reference and LTE as a tensor being tuned to the reference. Although we demonstrate the approaches for the special cases of STE and LTE, these approaches could be adapted to account for arbitrary b-tensor shapes.

#### 2.3.1 The concept of tuning in tensor-valued encoding

Different b-tensors can be considered*tuned*to each other if they yield approximately equal MD values also in the presence of TDD. Tuning helps eliminate the first order confounding effect of TDD by ensuringΔMD=0inEq. (3). The MD is derived from the first cumulant of the ensemble and direction average signal,

$$\langle \beta \rangle \approx b\;\text{MD}=b\;{\bar{D}}_{\text{iso}}\;=\frac{1}{\text{π}}\int_{0}^{\infty }s\left(\omega \right)\;{\bar{\lambda }}_{\text{iso}}\left(\omega \right)\text{ d}\omega ,$$ (15)

where${\bar{\lambda }}_{\text{iso}}(\omega )$is the average isotropic diffusion spectrum, defined as

$${\bar{\lambda }}_{\text{iso}}(\omega )=\frac{1}{3}\sum_{k=1}^{3}{\bar{\lambda }}_{k}(\omega ),$$ (16)

representing the average apparent*isotropic diffusivity*${\bar{D}}_{\text{iso}}$(Lundell & Lasič, 2020). The overbar denotes averaging over compartments with different eigen-spectra (Lasič et al., 2022).Eq. (15)highlights a key point: matching spectral traces is essential for tuning. We will demonstrate that this can be achieved through two different approaches.

#### 2.3.2 Tuning based on spectral projections and matching mean diffusivity

Different LTEs can be derived from STE by considering waveform projections

$$g_{\text{u}}\left(t\right)=\text{g}\left(t\right)\cdot \text{u}$$ (17)

with encoding power spectra$s_{\text{u}}(\omega )$fromEq. (12). For a powder sample, such LTEs would yield the attenuation factor as

$$\beta_{\text{u}}=b_{\text{u}}\;D_{\text{u}}=\frac{1}{\text{π}}\int_{0}^{\infty }s_{\text{u}}\left(\omega \right)\;{\bar{\lambda }}_{\text{iso}}(\omega )\text{ d}\omega .$$ (18)

The tuned LTE can be obtained by finding LTE projections yielding apparent diffusivity$D_{\text{u}}$equal to the MD from STE. This requires assuming a specific diffusion spectrum, for example, for spherical restrictions (seeFigs. 1Band2). To better suite hardware constraints, alternative projections, which sacrifice some tuning for reduced gradient amplitude, slew rate or their product,$\text{max}\left\{\left|g_{\text{u}}\right|\right\}\cdot \text{max}\left\{\left|{\dot{g}}_{\text{u}}\right|\right\}$, may be advantageous, as shown inFigure 2for the optLTE (Lasič et al., 2023).

**Fig. 2.**
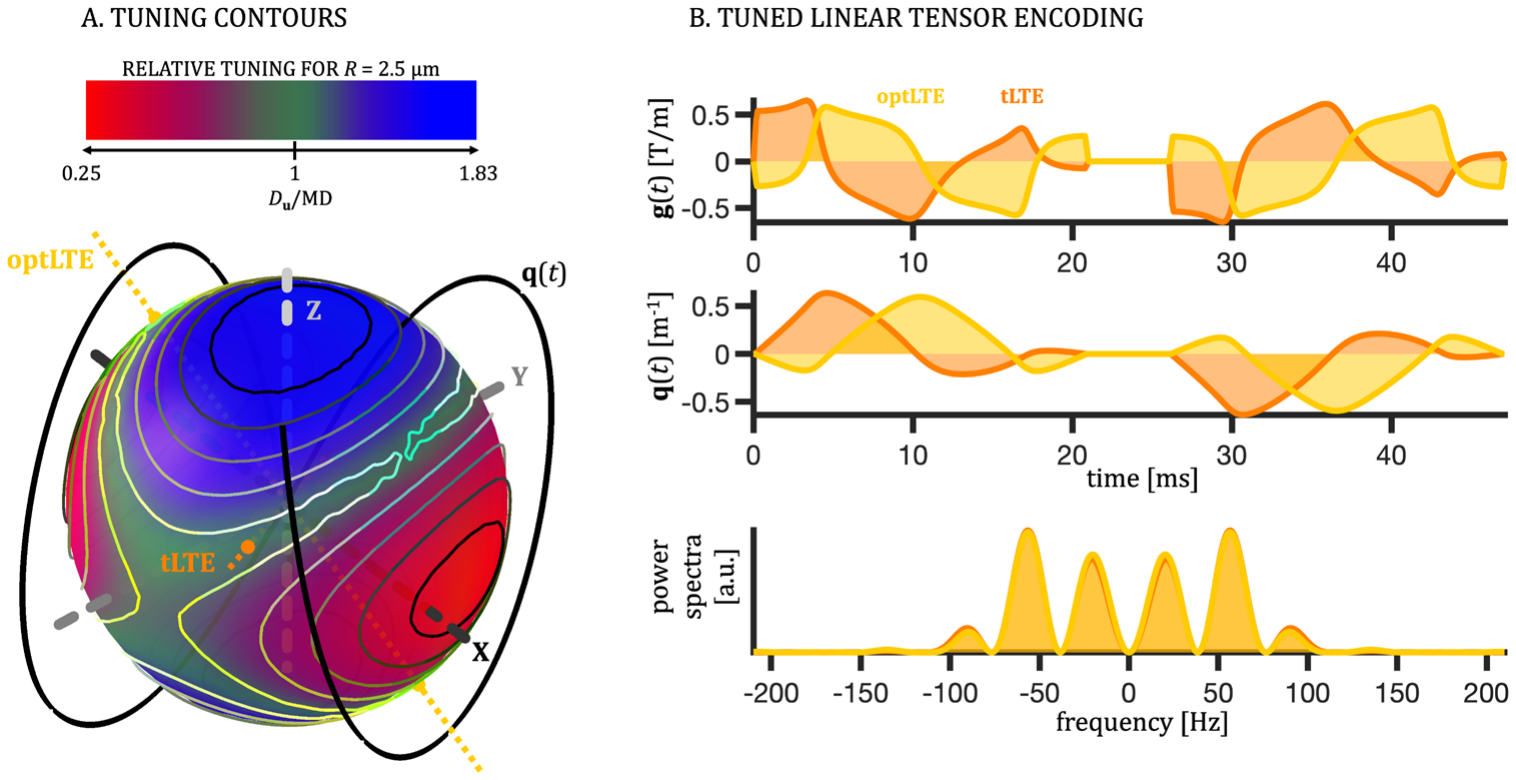
Tuning for spheres of*R*= 2.5 µm. (A) Color-coded relative tuning,$D_{\text{u}}\;/\;\text{MD}$, for spheres of*R*= 2.5 µm and*D*_0_= 2 µm^2^/ms. The LTE projection which minimizes the difference$\left|D_{\text{u}}-\text{MD}\right|$is shown in orange (tLTE). The additional “optimized” tuned LTE is shown in yellow (optLTE). This projection has the minimum product between maximum gradient magnitude and maximum gradient slew rate,$\text{max}\left\{\left|g_{\text{u}}\right|\right\}\;\;\cdot \;\;\text{max}\left\{\left|{\dot{g}}_{\text{u}}\right|\right\}$among 10% of best tuned projections. (B) The effective gradient and dephasing waveforms, and the corresponding power spectra for tLTE (orange) and optLTE (yellow). The optLTE has lower$\text{max}\left\{\left|g_{\text{u}}\right|\right\}$and$\text{max}\left\{\left|{\dot{g}}_{\text{u}}\right|\right\}$compared with tLTE, 583 mT/m and 1.3 T/ms/m versus 652 mT/m and 2.6 T/ms/m, respectively.

Such tuning approach does not aim to directly match the spectral traces (seeEqs. (11)and(15)), but rather to match the apparent mean diffusivities, which depends on both the encoding and diffusion power spectra. Although this does not represent a universal tuning strategy, the smooth nature of the encoding and diffusion spectra warrant relative robustness of the proposed approach, where tuning is approximately invariant in a wide range of restriction sizes (seeFig. 3).

**Fig. 3.**
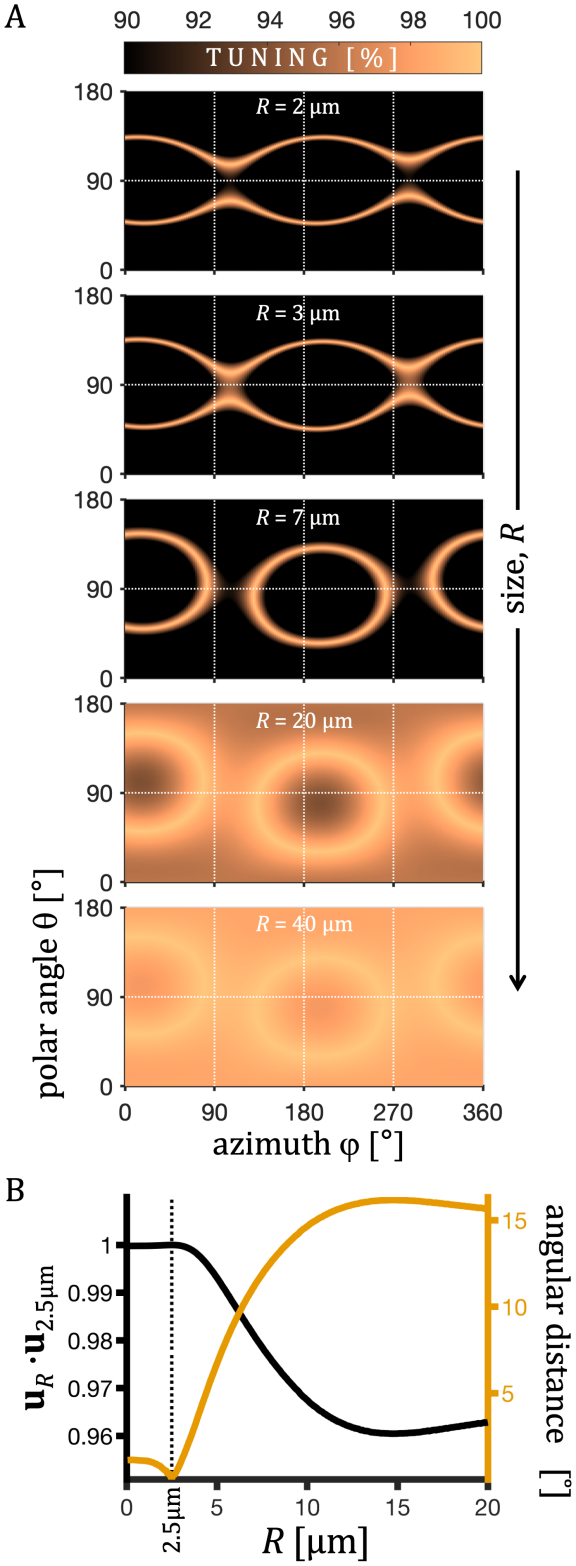
Tuning landscapes for spectral projections (LTE) from spherical tensor encoding (STE) at varying sphere sizes. (A) Tuning proximity$1\;-|1-D_{\text{u}}/\;\text{MD}|$is shown as a function of the polar and azimuthal angles of spectral projections from STE for sphere radii ranging from 2 to 40 µm. Hills and valleys along the angular coordinates indicate better and worse tuning, respectively. The position of the tuning contours is relatively independent of restriction size. For large spheres, the relative tuning$D_{\text{u}}/\;\text{MD}$remains close to 1, reflecting reduced TDD sensitivity as diffusion approaches the Gaussian regime for the given encoding timing. (B) The angle between the tuned projections ($D_{\text{u}}\;/\;\text{MD}=1$) for spheres of radius 2.5 µm (${\text{u}}_{2.5\;\text{µm}}$) and for spheres of radii*R*(${\text{u}}_{R}$), given as the angular distance∈(right axis) and as$\cos\in \;={\text{u}}_{R}\cdot {\text{u}}_{2.5\text{ µm}}\;$(left axis). The small angle variation for LTE projections across a wide range of sphere sizes suggests robust tuning for restricted diffusion.

#### 2.3.3 Tuning based on geometric averaging of signals

As we see fromEq. (15), given equal b-values, two otherwise different b-tensors are considered tuned if their spectral tracess(ω)are exactly matching (see panelsFigs. 1Aand2B). Such requirement is more stringent compared with matching MDs, but it ensures tuning independent of diffusion spectra. While matching spectral traces might be challenging, an equivalent tuning can be attained via geometrical averaging of signals from any orthogonal set of tensor projections. Consider a powder sample and the geometric/arithmetic$\left({\langle \cdots \rangle }_{\text{g}}/{\langle \cdots \rangle }_{\text{a}}\right)$averages of signals from LTE projections along orthogonal directions. Since the geometric average signal is in the first approximation given by the arithmetic average attenuation factor,

$$\frac{S_{\text{geo}}}{S_{0}}\approx {\langle e^{-\beta_{\text{u}}}\rangle }_{\text{g}}=e^{{\langle -\beta_{\text{u}}\rangle }_{\text{a}}},$$ (19)

the geometric average of signals from LTE projections corresponds to the signal encoded with the spectral trace and yields equal MD values. To match the encoding powers (b-values), the LTE gradient waveforms need to be appropriately scaled as$g_{\text{LTE}}(t)=c^{1/2}\;g_{\text{u}}(t)$, where$c=\frac{b}{b_{u}}=\frac{\int_{0}^{\infty }s\left(\omega \right)\;\text{d}\omega }{\int_{0}^{\infty }s_{u}\left(\omega \right)\;\text{d}\omega }$, which is equal to 3 when STE is the tuning reference. The geometric average signal inEq. (19)is expected to approximate well the tuned LTE signal up to the second cumulant (diffusion variance), assuming negligible effects of intra-compartmental kurtosis and exchange.

### 2.4 Spectral anisotropy (SA) and the spectral principal axis system (SPAS)

In addition to the tuning property, b-tensors generally exhibit varying sensitivities to TDD along different tensor axes (Lasič et al., 2021;Lundell et al., 2018;Lundell & Lasič, 2020;Teh et al., 2021), a property referred to as the*spectral anisotropy*(SA) (Lundell & Lasič, 2020). SA represents the directional variation in encoding power distribution, as defined inEq. (12). This can be visualized using color coding (Fig. 1), where the relative weights for red, green, and blue (RGB) are computed for each projection as$\frac{\int_{\omega \in \text{Ω}}s_{u}(\omega )\text{ d}\omega }{\int_{0}^{\infty }s_{u}(\omega )\;\text{d}\omega }$, whereΩrepresents the low-, mid-, and high-frequency bands. In the presence of TDD, SA may cause spherical tensor encoding (STE) to lose its rotational invariance, meaning it no longer yields truly isotropic diffusion encoding but instead depends on the relative orientation of the encoding (q-trajectory) and the diffusion compartment (Jespersen et al., 2019;Lasič et al., 2021). As a result, when averaging over orientations (powder averaging), SA can introduce additional variance of apparent diffusivities ($V_{D}$inEq. (1)) in samples with anisotropic structures, potentially biasing the assessment of microscopic anisotropy (Jespersen et al., 2019;Lasič et al., 2021;Lundell & Lasič, 2020;Lundell, Nilsson, Dyrby, et al., 2019;Lundell et al., 2018). The residual diffusion variance due to SA in STE is shown for simple restricted diffusion compartments under GPA inFigure 4D. The analysis of TDD in tensor-valued encoding was presented by Lundell and Lasič (Lundell & Lasič, 2020), and a summary of results for the second cumulant, highlighting the effect of SA, is provided in theSupplementary Materials.

**Fig. 4.**
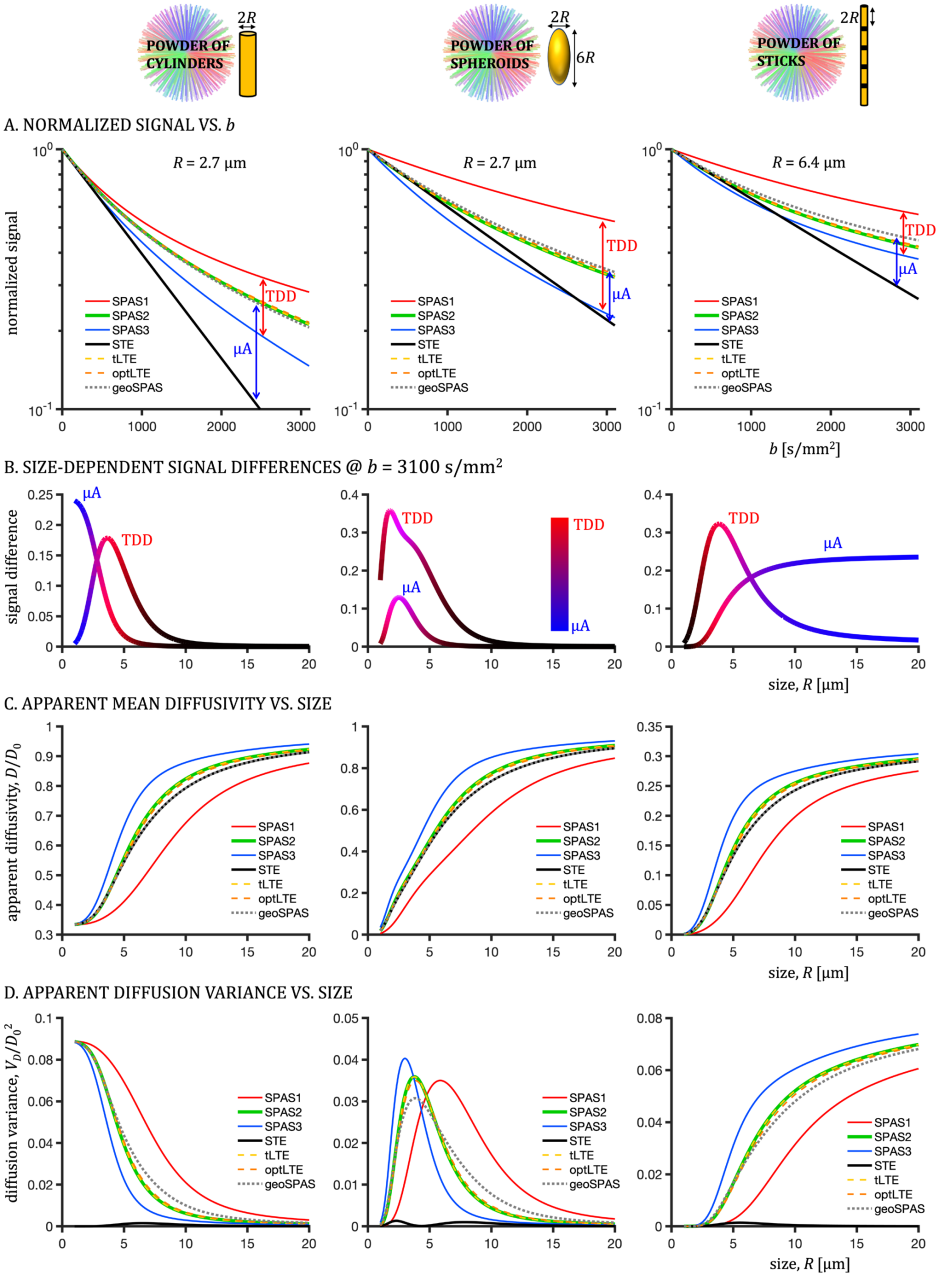
Theoretical predictions for restricted diffusion in powders of cylinders, spheroids, and sticks of finite length (left to right). (A) Normalized powder average signals versus b-value for SPAS1-3, STE, tLTE, optLTE, and for geometrically averaged SPAS LTEs (geoSPAS). Signals are shown for selected restriction sizes, such that the effects of both microscopic anisotropy (µA) and time-dependent diffusion (TDD) are clearly visible. Signals decrease from SPAS1 (low frequency) to SPAS3 (high frequency) for all substrates. The tLTE and optLTE were tuned for spheres of*R*= 2.5 µm and the corresponding signals approximate well the geoSPAS signals, which represent ideally tuned LTE to STE in terms matching spectral traces. Signal differences between geoSPAS and STE provide a contrast due to µA, while the difference between SPAS1 and SPAS3 are due to TDD. (B) Competing contrasts due to µA and TDD as a function of restriction size. Shown are signal differences at b-value of 3100 s/mm^2^between geoSPAS and STE (µA, blue) and between SPAS1 and SPAS3 (TDD, red). The effect of TDD is pronounced at intermediate sizes for all substrates, while µA contrast decreases with size for cylinders and increases with the length of sticks (zero radial and restricted axial diffusivity). The case of spheroids exhibits more complex behavior due to the competing effects along the long and short restriction axes (size ratio 1:3). (C) Normalized powder average apparent mean diffusivity as a function of restriction size. The diffusivities increase for SPAS1 to SPAS3 encodings. A good correspondence between the geoSPAS, tLTE, and optLTE can be appreciated for a wide range of sizes. Small differences between geoSPAS and the tuned encodings occur at intermediate sizes (about factor 4 larger than the tuning size of 2.5 µm). (D) Normalized powder average variance of apparent diffusivities,$V_{D}$, as a function of restriction size. The ordering of$V_{D}$from SPAS1 to SPAS3 depends on the substrate. While the effects of higher orders in b were ignored in this simulation, it is noteworthy that the first order effects propagate to higher b-values, such that consistency in$V_{D}$between geoSPAS and tuned encodings is approximately maintained. The small diffusion variance from STE at intermediate sizes is due to spectral anisotropy as noted previously using Monte-Carlo simulations (see supplementary information inLundell, Nilsson, Dyrby, et al., 2019).

We define the*spectral principal axis system*(SPAS) as the orthogonal set of directions that exhibit maximally different sensitivities to TDD. Because TDD effects arise from the interaction between the encoding and diffusion spectrum, TDD sensitivity can only be defined in relation to the diffusion spectrum. This implies that SPAS does not represent a universal encoding property, as its definition is context dependent. Although SA can be fully described by considering all the spectral components of$s_{\text{u}}(\omega )$or by analyzing the b-tensor spectrum—that is, breaking down the b-tensor into contributions from different frequency channels (seefigure 2.13BinLundell & Lasič, 2020)—the SPAS offers an approximate, yet practical, approach for evaluating SA.

#### 2.4.1 SPAS from low-pass filtered b-tensor

To gauge TDD differences for different spectral projections$s_{\text{u}}(\omega )$, we split the entire frequency range into three bands with equal encoding power. The crossover frequencies were determined based on the spectral traces(ω), such that the total cumulative power,

$$b(\omega )=\frac{1}{\text{π}}\int_{0}^{\omega }s(\omega^{\prime })\text{ d}\omega^{\prime },$$ (20)

reaches 1/3 and 2/3 of the total b-value (seeFig. 1BandEq. (10), c.f.figure 2.15inLundell & Lasič, 2020). The spectral bands each contain one-third of the total encoding power, and their exact spectral content is not critical. The gradual transition of encoding power across directions (seeEq. (12)) ensures that when one band is prominent, the others contribute less. The SPAS can be defined ad hoc simply as the eigenvectors of the low-pass filtered b-tensor, that is, the tensor resulting from setting the integration limits inEq. (9)as the crossover frequency of the low-frequency band. For waveforms with pronounced SA, this results in distinct spectral projections along the eigenvector axes of the low-pass-filtered b-tensor, capturing different TDD sensitivities, as shown inFigure 1B. Although RGB is used for both spectral anisotropy weighting and SPAS projections, this does not imply a one-to-one correspondence. SPAS projections are not band limited but span the full frequency range with overlapping spectra (Fig. 1C), each emphasizing different TDD sensitivities. In general, waveforms optimized for efficient b-weighting tend to concentrate encoding power at lower frequencies. The q-MAS serves as an illustrative example of an isotropic b-tensor, whereq(t)oscillates with high frequency orthogonal to a low-frequency (PGSE-like) axis (Lasič et al., 2014;Lundell, Nilsson, Dyrby, et al., 2019;Topgaard, 2013). The SPAS approach assumes spectral anisotropy in the low-frequency band for meaningful separation of TDD sensitivities. Experimentally feasible waveforms naturally exhibit sufficient spectral anisotropy to ensure distinct spectral projections. In principle, if a waveform exhibited a recessed mid-range along at least one projection—where both low- and high-frequency bands are strong—SPAS may misrepresent the directional variation of TDD sensitivity. The SPAS approach may not be relevant in cases with generally low SA, such as for multiple rotations of the q-vector (H. Jiang et al., 2023), where TDD sensitivity is more evenly distributed.

#### 2.4.2 Alternative derivation of SPAS based on encoding spectral moments

Alternatively, the SPAS could be defined in terms of the encoding spectral moments (see chapter 2.5.9 inLundell & Lasič, 2020),

$$m_{ij}^{\left(p\right)}=\frac{1}{\text{π}}\int_{0}^{\infty }s_{ij}(\omega )\;\omega^{p}\text{ d}\omega ,$$ (21)

which for*p*= 1,2… corresponds toEq. (13)in the low-frequency approximation of the diffusion spectrum, applicable when encoding comprises power at relatively low frequencies (Lundell & Lasič, 2020). The case of*p*= 1 could, for example, apply to extracellular hindered diffusion (Novikov et al., 2011,^2014^;Novikov, Fieremans, et al., 2018), whereD(ω)∝|ω|, while the case of*p*= 2 could be suited for restricted diffusion, where$D(\omega )\propto \omega^{2}$and$m_{ij}^{\left(2\right)}$projections represent spectral variance (Burcaw et al., 2015;Chakwizira, Westin, et al., 2023;Lasič et al., 2006,^2024^;Lundell & Lasič, 2020;Nilsson et al., 2017;Stepišnik, 1993;Stepišnik et al., 2006).

The eigenvectors of$m_{ij}^{\left(p\right)}$could be used to provide alternative SPAS versions. In contrast to the low-pass filtered encoding spectrum used inEqs. (20)and(21), the spectral moments involve high-pass filtering. However, the SPAS axes derived from the low-pass and high-pass filtered encoding spectra are expected to be nearly equivalent, provided that the high- and low-frequency axes of the SPAS are swapped (Supplementary Fig. S1). The equivalence or complementarity of the SPAS from such different approaches is a consequence of low- and high-frequency contributions summing up to the full b-tensor.

#### 2.4.3 Centroid encoding frequencies

The centroid encoding frequency has been used in oscillating gradient experiments with LTE (Arbabi et al., 2020) and adopted for tensor-valued encoding (Narvaez et al., 2022), defined as

$$\omega^{\text{c}}=\frac{\int_{0}^{\infty }s(\omega )\;\omega \text{ d}\omega }{\int_{0}^{\infty }s(\omega )\;\text{d}\omega }$$ (22)

Similarly, the “projected” centroid frequency could be defined for b-tensor projections along vectorsuas

$$\omega_{u}^{\text{c}}=\frac{\int_{0}^{\infty }s_{u}(\omega )\;\omega \text{ d}\omega }{\int_{0}^{\infty }s_{u}(\omega )\;\text{d}\omega }$$ (23)

Eq. (23)could be applied along the SPAS axes to yield the corresponding “projected” centroid frequencies. Note that$\omega_{\text{u}}^{\text{c}}$are not defined along the$m_{ij}^{\left(p\right)}$eigenvectors with zero eigenvalues (seeEq. (21)). Importantly, for the SPAS,$\omega_{\text{u}\;=\;\text{SPAS}}^{\text{c}}$provides a measure of the spread of encoding frequencies, which could be used to gauge SA. Reduced variation in$\omega_{\text{u}}^{\text{c}}$reflects reduced SA, where in the limit of spectrally isotropic encoding (SA = 0), the variation in$\omega_{\text{u}}^{\text{c}}$vanishes and$\omega_{\text{u}}^{\text{c}}=\omega^{\text{c}}$. SA generally decreases with increasing degrees of motion nulling, for example, by repeating encoding over several periods, resulting in no encoding at zero frequencies (Fig. 1). This is similar to the narrowing of the frequency window for oscillating gradients (Callaghan & Stepišnik, 1996) or using double rotation of the q-vector (H. Jiang et al., 2023). The limit case of SA = 0 is practically unattainable since infinite encoding frequencies would be required, corresponding to an infinite number of q-trajectory excursions that would eventually even out any directional differences in encoding power spectra.

## 3 Methods

### 3.1 Gradient waveforms

The STE was obtained using the Matlab (The MathWorks, Natick, MA) package NOW (https://github.com/jsjol/NOW) for numerical optimization of tensor-valued encoding waveforms under given hardware constraints (Sjölund et al., 2015). Matlab code for generating gradient waveforms used in this work is available athttps://github.com/samo-lasic/Lasic_SPAS_ImagingNeuroscience2025. To avoid concomitant field effects and obtain velocity compensation, we repeated the STE waveforms after the inversion RF pulse (seeFig. 1A;Szczepankiewicz, Westin, et al., 2021). The SPAS was obtained as eigenvectors of the low-frequency filtered STE as described inSection 2.4and illustrated inFigure 1B. The low-frequency band was determined from the spectral trace yielding 1/3 of the total encoding power (b-value). The LTE projections corresponding to SPAS are shown in panels B and C ofFigure 1as SPAS1, SPAS2, and SPAS3. Signals from these waveforms were geometrically averaged to obtain the geoSPAS dataset (Section 3.4).

The tuned LTEs were obtained as described inSection 2.3.2by taking 1D projections (LTE) of the 3D waveform (STE) along 1000 uniformly distributed directions, and finding the 1D waveform that yields apparent diffusivity$D_{\text{u}}$value most similar to MD from STE for spheres of radius*R*= 2.5 µm and bulk diffusivity*D*_0_= 2 µm^2^/ms. The MD was calculated byEqs. (11)and(15), and$D_{\text{u}}$byEqs. (12)and(18). From 10% of best tuned LTE projections shown inFigure 2, the one minimizing the difference$\left|D_{\text{u}}-\text{MD}\right|$was chosen as the tuned LTE (tLTE), while the “optimized tuned” LTE (optLTE) (Lasič et al., 2023)) was chosen as the one with the lowest demand on the gradient system, defined as the one minimizing the product of maximum gradient magnitude and maximum gradient slew rate,$\text{max}\left\{\left|g_{\text{u}}\right|\right\}\cdot \text{max}\left\{\left|{\dot{g}}_{\text{u}}\right|\right\}$.

The gradient waveforms were played in two equal 21 ms long encoding blocks separated by a 5 ms gap for the refocusing RF pulse. The waveforms were copied on both sides of the refocusing pulse, yielding velocity-compensated effective gradient waveforms of total duration 47 ms as shown inFigures 1Cand2B. Full velocity compensation ensures no encoding of incoherent ballistic flow, which can otherwise confound microstructural assessments due to pseudo-diffusion*in vivo*(Ahlgren et al., 2016). The centroid frequencies were$\omega_{ij}^{\text{c}}/\;2\pi$= 45 Hz for the STE and in the range 21–69 Hz for SPAS-LTEs. The exchange weighting times (Ning et al., 2018) were in the range Γ = 1.6–1.8 ms.

### 3.2 Simulations

Simulations were implemented in Matlab (The MathWorks, Natick, MA). Code is available athttps://github.com/samo-lasic/Lasic_SPAS_ImagingNeuroscience2025. Apparent diffusion coefficients (ADC) were calculated according toEqs. (13)and(14)using expressions for diffusion restricted in spheres, cylinders, and planes (Lundell & Lasič, 2020;Stepišnik, 1993) with bulk diffusivity of*D*_0_= 2 µm^2^/ms.

Axisymmetric spheroids were modeled by the diffusion spectrum for spheres with two different restriction sizes. This approximation has been previously used and validated by simulations (Lasič et al., 2022;Lundell, Nilsson, Dyrby, et al., 2019;Nielsen et al., 2018). Diffusion in sticks of finite length and zero radial diffusivity was modeled using the diffusion spectrum for planar geometry. ADCs were obtained for 300 uniformly distributed rotations and average signals resulted from the arithmetic average of the corresponding 300 monoexponentially attenuated signals (Fig. 4A). Note that rotating the substrate is equivalent to rotating the encoding. This was performed for all 6 waveforms and for 100 restriction radii linearly spaced in the range*R*= 1–20 µm.

### 3.3 Experiments

All experiments were approved by the Danish Animal Experiments Inspectorate (2018-15-0201-01551) following the European Communities Council Directive (2010/63/EU).*Ex vivo*and*in vivo*rat brains as well as phantom data were acquired on a Bruker BioSpec 7T (Bruker BioSpin, Ettlingen, Germany) MR Scanner with 0.66 T/m gradients. The individual protocols are described below.

#### 3.3.1 Ex vivo rat brain

Perfusion-fixed Sprague-Dawley adult rat brains stored at 4°C in paraformaldehyde (PFA) were transferred to a glass tube filled with a fluorocarbon fluid (Fluorinert, Sigma-Aldrich, Germany). After 2 to 3 h at room temperature (22 ± 1°C), the sample was secured in the Bruker mouse cryoprobe cradle and positioned at the center of the magnet. The sample temperature was stabilized at 27°C using both the surface temperature sensor control of the cryo-coil and a heated airflow with an additional temperature probe attached underneath the sample outside the field of view (SA Instruments, New York, USA). Temperature deviations recorded with the additional probe were within 0.1°C during the experiment. A mouse^1^H quadrature T/R cryoprobe surface array coil was used for measurements (Bruker, Germany).

#### 3.3.2 In vivo rat brain

One Sprague-Dawley female rat (n = 1; 180 g) was continuously anesthetized using 2–3% isoflurane in air and oxygen. The rat head was secured in a stereotactic frame using a bite bar and ear bars. Respiratory rate was monitored through a pillow placed underneath the rat’s body, and temperature was maintained at 37.5 ± 0.5°C using a rectal probe connected to warmed air blower (SA Instruments, New York, USA). An 86-mm diameter volume coil and 20 mm diameter single-loop surface coil (Bruker, Ettlingen, Germany) were used for transmission and reception, respectively.

#### 3.3.3 Phantoms

A 1 cm plastic tube filled with demineralized water was used to test the calibration of the gradients for all the encoding waveforms and imaged with the same setup as for the*ex vivo*sample.

#### 3.3.4 Diffusion weighted MRI

A multi-shot echo planar imaging sequence customized for diffusion encoding with general gradient waveforms (https://osf.io/t9vqn/) was used. Both*in vivo*and*ex vivo*scans used TR/TE = 3 s/53.136 ms, matrix size = 96 x 96 and 6 axial slices of 1 mm thickness. Adjustments included acquisition of a B0-field map and global shimming with MAPSHIM, along with EPI-ghost and trajectory corrections. Diffusion encodings were performed using 5 waveforms (STE, SPAS1, SPAS2, SPAS3, tLTE) with 12 rotations corresponding to the vertices of the icosahedron.

*Ex vivo scans*used 16 segments per image, 6 averages, FOV = 16 x 16 mm^2^, in-plane resolution = 0.17 mm^2^, scan time = 48.4 h, 10 logarithmically spaced b-values (123, 168, 257, 366, 550, 805, 1194, 1760, 2560, 3806 s/mm^2^).

*In vivo scans*used 4 segments per image, 3 averages, FOV = 25 x 25 mm^2^, in-plane resolution = 0.26 mm^2^, scan time = 3.65 h, 6 logarithmically spaced b-values (123, 238, 466, 952, 919, 3806 s/mm^2^).

### 3.4 Data analysis

Preprocessing steps included Marchenko–Pastur denoising implemented in Matlab (https://github.com/sunenj/MP-PCA-Denoising) (Does et al., 2019;Veraart et al., 2016) and extrapolation-based motion and eddy-current correction (Nilsson et al., 2015). Data analysis was performed with Matlab using a combination of custom scripts (https://github.com/samo-lasic/Lasic_SPAS_ImagingNeuroscience2025) and parts of the multidimensional diffusion MRI framework (https://github.com/markus-nilsson/md-dmri). Smoothing was applied per slice with 2D Gaussian smoothing kernel with standard deviation of 0.5. Signal attenuations from SPAS-LTEs were geometrically averaged, yielding the geoSPAS dataset. To estimate diffusion tensors, a subset of geoSPAS data acquired with 12 waveform directions and b-values in the range 123–805 s/mm^2^were used. Nonlinear least squares fitting was applied with symmetric positive-definite constraints to yield maps of signal without diffusion weighting (*S*_0_), mean diffusivity (MD), and fractional anisotropy (FA). To generate maps of microscopic fractional anisotropy (µFA), data from STE and geoSPAS were averaged across 12 directions, and constrained nonlinear least squares fitting was applied using the gamma function as described previously (Lasič et al., 2014).

Model-free contrast maps associated with microscopic anisotropy (µA) and time-dependent diffusion (TDD) were obtained by subtraction of signals from different encodings (seeEq. (3)). Logarithms of direction averaged signals (log[〈S〉]) were taken at the maximum b-value of 3806 s/mm^2^. The differences (Δlog[〈S〉]) between geoSPAS and STE were used to generate the µA contrast maps according to

$$\text{µA}=\log{\left[\langle S\rangle \right]}_{\text{geoSPAS}}-\log{\left[\langle S\rangle \right]}_{\text{STE}}$$ (24)

and the differences between SPAS1 and SPAS3 were used for the TDD contrast maps according to

$$\text{TDD}=\log{\left[\langle S\rangle \right]}_{\text{SPAS}1}-\log{\left[\langle S\rangle \right]}_{\text{SPAS}3}.$$ (25)

Subtracting logarithms of signals instead of raw signals yields contrast related to signal attenuation, effectively removing the b0 signal contribution. Regions of interests (ROIs) where drawn manually by reference to a rat brain atlas (Paxinos & Watson, 1996) in the cortical gray matter (CGM), choroid plexus (CP), dentate gyrus of the hippocampus (DGHC), and white matter (WM). Additional ROIs were generated by segmentation based on the ranges of µA and TDD contrasts (Supplementary Fig. S4). ROI-average signals versus b-value were normalized using*S*_0_extrapolation from the Gamma distribution fit of the multiexponential signal attenuations (Lasič et al., 2014). Full signal representations in the presence of restricted diffusion are derived in theSupplementary Materials.

## 4 Results

### 4.1 Tuning

STE waveforms (gradient, dephasing) and the spectral trace are shown inFigure 1A. The nonexistent zero-frequency component shows that the encoding is velocity compensated. The rotation relative to the laboratory frame (XYZ axes) is arbitrary and depends on the output from the optimization (Sjölund et al., 2015). Thus, using any of the XYZ projections for tuning purposes is generally not applicable, even though such approach can be justified as a proxy for tuning when specific q-trajectories are used (Lundell, Nilsson, Dyrby, et al., 2019). Spectral anisotropy of the STE is shown inFigure 1B, color coded with red, green, and blue, based on the relative amounts of encoding power from the low-, mid-, and high-frequency bands for each spectral projection. The crossovers between the frequency bands were determined from spectral trace yielding 1/3 and 2/3 of total encoding power (b-value), as depicted in panel B. The SPAS, shown inFigure 1Bas red, green, and blue lines, corresponding to the eigenvectors of the low-pass filtered b-tensor, maximizes the spread of encoding power between the low-, mid-, and high-frequency bands. This is shown inFigure 1B and Cby comparing the encoding power spectra for the SPAS1-SPAS3 LTE waveforms.

The tuning is shown inFigure 1Bas contours outlining 10% of all LTE projections with smallest values of$\left|D_{\text{u}}-\text{MD}\right|$for spheres of*R*= 2.5 µm (solid lines) and*R*= 5 µm (dashed lines). For spheres of*R*= 2.5 µm, tuning is also shown inFigure 2Aas the color-coded ratio$D_{\text{u}}/\;\text{MD}$. The tLTE refers to the STE waveform projection with$D_{\text{u}}\;=\text{MD}$. As an alternative to the tLTE, the “optimized tuned” LTE (optLTE) is included inFigure 2. In this case, the tuning was slightly compromised to reduce the maximum gradient magnitude from 652 mT/m to 583 mT/m and slew rate from 2.6 T/ms/m to 1.3 T/ms/m, respectively. Due to the smooth nature of the encoding and diffusion spectra, the goal of matching MDs is equivalent to matching the envelopes of encoding spectra from LTE and the spectral trace from STE. The robustness of such tuning approach can be appreciated by comparing the tuning contours for two restriction sizes (solid and dashed lines), shown inFigure 1B, and noting that the tuned LTE projection shown inFigure 2Aapproximately corresponds to the intersection between the two contour lines. The tuning landscapes shown inFigure 3illustrate how tuning varies with spherical angle across different sphere sizes, showing how closely the relative tuning approaches the ideal value of 1. For larger spheres, tuning remains close to 1 across the landscape, reflecting reduced sensitivity to TDD as diffusion approaches the Gaussian regime. Smaller spheres exhibit greater modulation, with sharper contours, while for very small structures, tuning becomes less relevant as both MD and$D_{\text{u}}$approach 0 (c.f.Fig. 4). Notably, the position of the tuning contours is largely independent of sphere size, indicating robust tuning across a wide range of restriction sizes.

### 4.2 Simulations

The competing contrasts due to µA and TDD were simulated for diffusion within powders of cylinders, spheroids, and sticks of finite length (seeFig. 4). Signals versus b-value are shown for selected restriction sizes, for which both contrasts are visible. The encoding power gradually shifts from lower to higher frequencies for SPAS1 to SPAS3 encodings (Fig. 1), resulting in decreasing signals (Fig. 4A) and increasing ADC values (Fig. 4C) due to TDD. The tLTE and optLTE yield similar signals, which are between SPAS1 and SPAS3 and approximately match the geometrically averaged SPAS signals (geoSPAS). Note that the geoSPAS signals represent ideal matching of spectral traces and thus ideal tuning. While signal attenuations from LTEs are multi-exponential due to orientation dispersion, signals from STE are approximately mono-exponential due to mono-dispersed restriction size. The initial slope and the deviation from mono-exponential signal attenuation are related to the mean apparent diffusivity and the diffusion variance, respectively, shown in panels C and D. The signal difference between geoSPAS and STE at high b-values indicates the effect of µA unconfounded by restricted diffusion. The size dependence of TDD and µA is shown inFigure 4B. For cylinders, the µA contrast is maximized for small sizes, when diffusion appears Gaussian, similar as for example in hexagonal liquid crystals (Lundell, Nilsson, Dyrby, et al., 2019). With increasing cylinder radius, the radial ADC increases and the µA contrast decreases. The TDD contrast peaks at intermediate sizes, depending on encoding times, and fades away for large sizes when diffusion again appears Gaussian (compare panels B and C). For sticks, the size dependence of the TDD contrast is similar as in the case of cylinders, but the µA contrast exhibits the opposite trend. For shorter sticks, the axial ADC decreases toward the radial ADC of zero, while it is maximized for large sizes, leading to maximum µA contrast for longer sticks. A more complex behavior can be seen for the spheroids. As in the case of cylinders, both contrasts decrease for large sizes as radial and axial ADCs approach bulk diffusivity ($D_{0}$). The TDD contrast is dominating due to the low compartment anisotropy and restriction along all axes (ratio 1:3). Two different restriction lengths result in a wider TDD peak compared with the cylinders. The µA contrast peaks at intermediate sizes and vanishes for small and large sizes, when diffusion appears Gaussian and ADCs are, respectively, zero and bulk ($D_{0}$). SA in STE leads to small deviations from mono-exponential decays. This is reflected also inFigure 4Das non-zero values of the diffusion variance$V_{D}$(black solid lines) at intermediate restriction sizes for all three substrates. However, the maximum$V_{D}$from STE was only about 3% of the maximum$V_{D}$from LTE across all the substrates. The effects of SA for restricted diffusion are analyzed in more detail in theSupplementary Materials.

### 4.3 Experiments

Reference experiments were conducted on water, confirming that the signals from all encodings overlap at equal b-values (Supplementary Figs. S2 and S3).*Ex vivo*results are shown inFigures 5 to 8and*in vivo*results are shown inFigures 9and10. Note that the ex vivo results were obtained with a cryo-coil optimized for the mouse brain. This setup maximized overall SNR, but the coil’s sensitivity profile tapers off in deeper brain regions.

**Fig. 5.**
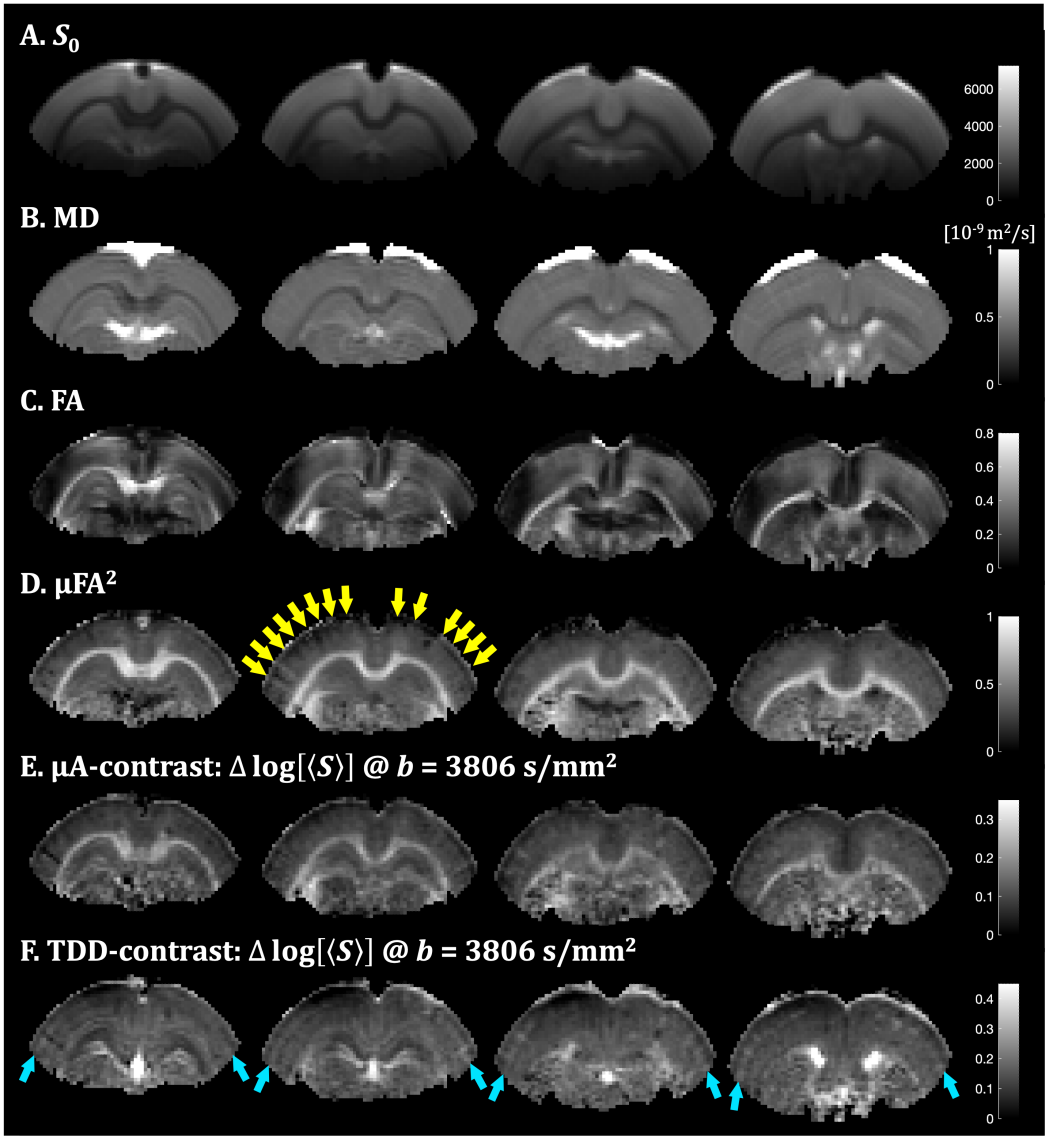
Contrast and parameter maps from the fixed rat brain measured with STE and SPAS encodings. The four axial slices are ordered from posterior to frontal (left to right). Parameter maps (A–D) were generated from STE and geoSPAS data. The*S*_0_, MD, and FA maps resulted from the DTI fit and the µFA map from the constrained gamma fit of direction average signals. The µA- and TDD-based contrast maps (E, F) were obtained by subtracting logarithms of direction-averaged signals (ln[〈S〉]) at b-value of 3806 s/mm^2^for SPAS1 and SPAS3 data and for geoSPAS and STE data, respectively. The yellow arrows indicate a radial pattern on the µFA map (D) and the bright blue arrows indicate tangential layers on the TDD map (F).

Signal attenuations from geometrically averaged SPAS LTEs (geoSPAS) yield*S*_0_, MD, and FA parameter maps shown inFigure 5A–C. Rotationally averaged geoSPAS and STE data yield µFA maps unconfounded by restricted diffusion (Fig. 5D). The µFA^2^maps approximately correspond to the µA-based contrast maps shown inFigure 5F, obtained from high b-value geoSPAS and STE signals as a subtraction map (Eq. (24)). The protocol unfolds the µA contrast from the TDD contrast, which is shown inFigure 5Eas a subtraction map of high b-value SPAS1 and SPAS3 signals (Eq. (25)). The relationship between TDD and µA contrasts is shown in the color-coded TDD-µA joint contrast maps inFigure 6. A closer look at the regions with cortical gray matter (CGM), choroid plexus (CP), dentate gyrus of the hippocampus (DGHC), and the white matter (WM) is shown inFigure 7. The relationship between µA and TDD contrasts can be visualized as a voxel-wise scatter plot inFigure 7Afor the different regions outlined inFigure 7B. Overall, the µA contrast is pronounced in the white matter and TDD contrast is higher in the CP and in the DGHC. The normalized direction and ROI-average signals are shown inFigure 7C. The normalized ROI signals consistently decrease for SPAS1 to SPAS3 as expected from theoretical predictions in the presence of restricted diffusion (Fig. 4). Importantly, the signals from the tuned LTE (tLTE) coincide well with the signals from the geoSPAS. In CGM, both contrasts are relatively low. However, an interesting heterogeneity is visible within CGM with µFA exhibiting radial patterns (Fig. 5D) and TDD showing a bright tangential layer (Fig. 5F).

**Fig. 6.**
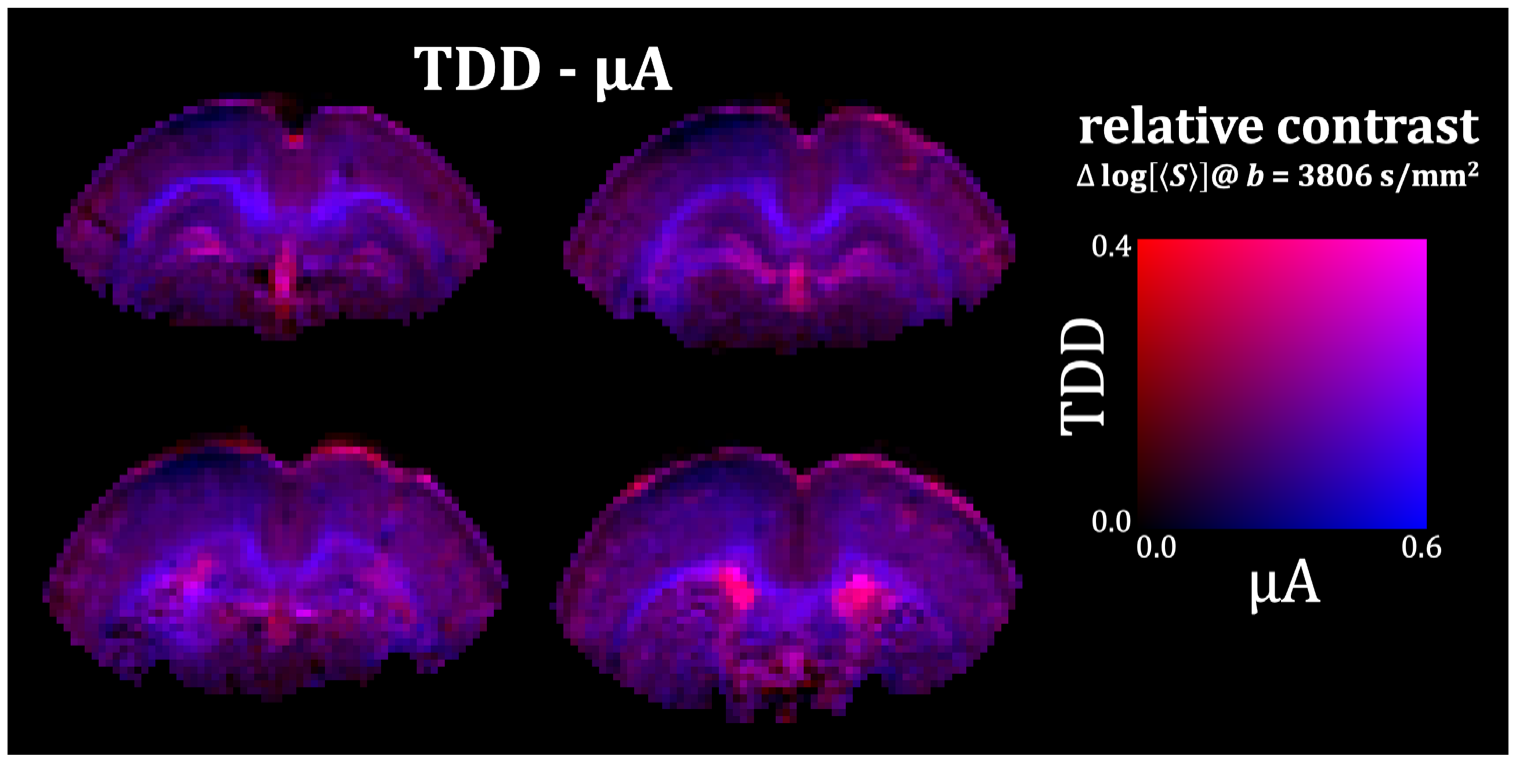
TDD-µA joint contrast map from the fixed rat brain. The separate contrasts from µA and TDD (shown inFig. 5E, F) are combined into a single color-coded map, with blue representing µA and red representing TDD. The relative contrasts are scaled to 60% and 40% of the µA and TDD contrast ranges, respectively. This map highlights tissue regions with distinct combinations of anisotropy (µA) and size-related (TDD) properties, providing an integrative view of microstructural differences.

**Fig. 7.**
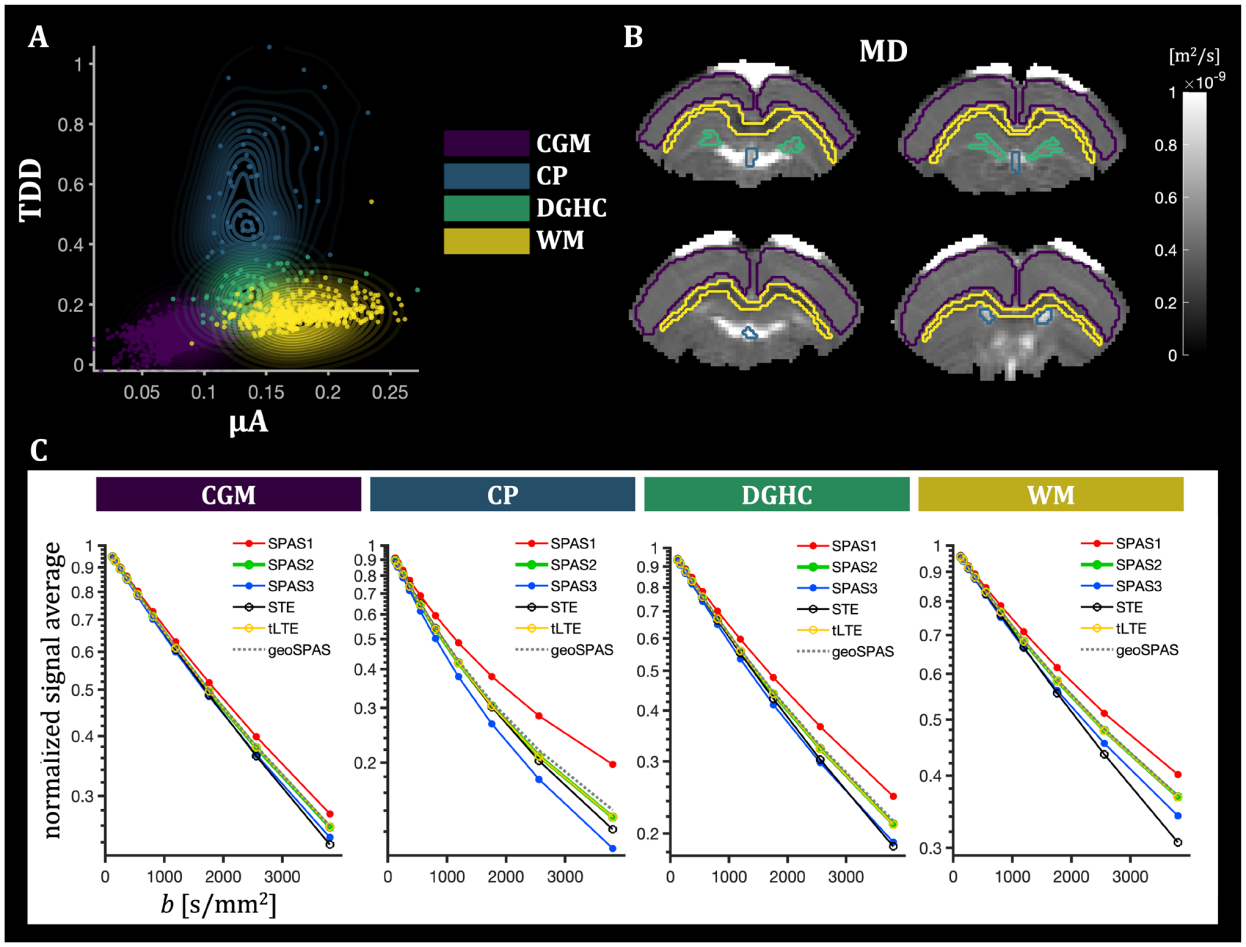
Voxel-wise scatter plot of μA and TDD contrasts (A), ROI selection (B), and ROI-average signals (C) for different regions of the fixed rat brain. The regions outlined on the MD maps include*cortical gray matter*(CGM),*choroid plexus*(CP),*dentate gyrus of the hippocampus*(DGHC), and the*white matter*(WM). The normalized ROI signals consistently decrease for SPAS1 to SPAS3 as expected from theoretical predictions in the presence of restricted diffusion. The signals from the tuned LTE (tLTE) coincide well with the signals from the geoSPAS. In CGM, both contrasts are relatively low. In WM, µA is large and the TDD contrast is relatively low but larger than in CGM. The CP and the DGHC exhibit intermediate µA and elevated TDD contrast, with pronounced TDD contrast in the CP. The STE signal is close to mono-exponential in the CGM and WM, and more multi-exponential in the CP and DGHC, indicating larger tissue heterogeneity in those regions. The pronounced deviation from mono-exponential STE signals in the CP is reflected also by the larger spread of the TDD contrast in this region.

In WM, µA is large and TDD is relatively low but larger than in CGM. The CP and the DGHC exhibit intermediate µA and elevated TDD contrast, with pronounced TDD in the CP. The STE is close to mono-exponential in the CGM and WM, and more multi-exponential in the CP and DGHC, indicating larger tissue heterogeneity in those regions. The pronounced deviation from mono-exponential STE signals in the CP is reflected also by the larger spread of the TDD contrast in this region. The LTE signals in the CP resemble the simulated scenario with spheroidal restrictions (Fig. 4A), where TDD is pronounced due to restrictions along all three axes, which could be related to the high density of cuboidal epithelial cells, fibroblasts, or macrophages (Saunders et al., 2023).

The effects of TDD on the µFA parameter maps from the fixed tissue when using the geoSPAS signals (geometric averaging) or not tuned SPAS LTE signals are shown inFigure 8. This figure demonstrates the extent of µFA bias that arises when waveforms with different TDD sensitivities are used, highlighting the importance of tuning to achieve unbiased µFA estimation. The geoSPAS yields the “correct” µFA map not confounded by restricted diffusion. In comparison, using SPAS3 and SPAS1 data results in reduced and elevated µFA, respectively. Note that the fitting model used to estimate µFA assumes that STE and LTE yield equal MD values, an assumption that was not fulfilled for the maps inFigure 8A and C. The decreased/increased µFA for SPAS3/SPAS1 is due to increased LTE signal differences relative to the STE signal. The µFA difference maps reflect the effects of TDD (D-E). The fitting noise propagates from µFA with SPAS3 to the differences in D. The µFA difference from SPAS1 and geoSPAS (E) is more accurate, and this map resembles the TDD-based contrast maps inFigure 5Fobtained by subtracting logarithms of direction-averaged signals. However, additional contrast is visible on the µFA difference (∆µFA^2^) inFigure 8E(slice 2) between the DGHC and the WM, and the layers in CGM are more pronounced than inFigure 5F. Additional µFA parameter maps can be found in theSupplementary Figure S5.

**Fig. 8.**
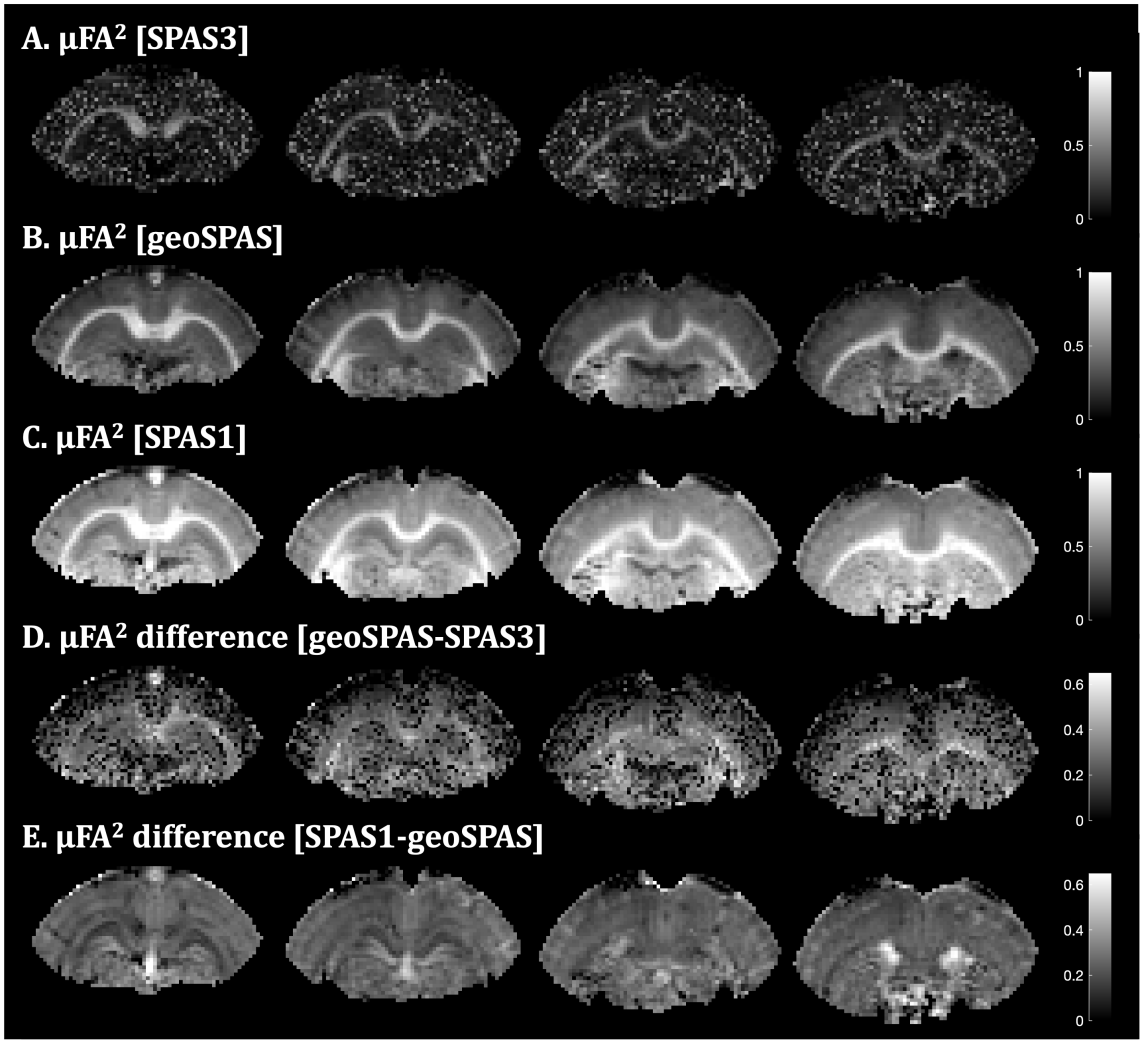
Correct and biased µFA parameter maps from the fixed rat brain estimated from geoSPAS and not tuned SPAS LTE signals. Using geoSPAS yields the “correct” µFA map not confounded by TDD (B). Reduced signal difference between STE and SPAS3, caused by TDD, results in lower µFA and reduced precision (A). The µFA increases for SPAS1 due to increased signal differences relative to the STE signal (C). The µFA difference maps reflect the effects of TDD (D–E). Fitting noise propagates from µFA with SPAS3 (A) to the differences in panel D. The µFA difference from SPAS1 and geoSPAS (E) is more accurate, and this map is similar to the TDD-based contrast maps inFigure 5Fobtained by subtracting logarithms of direction-averaged signals. However, some regions are more clearly delineated on the µFA difference in panel E (slice 2), such as the dentate gyrus of the hippocampus and the cortical layers. Additional µFA parameter maps are provided in theSupplementary Figure S5.

**Fig. 9.**
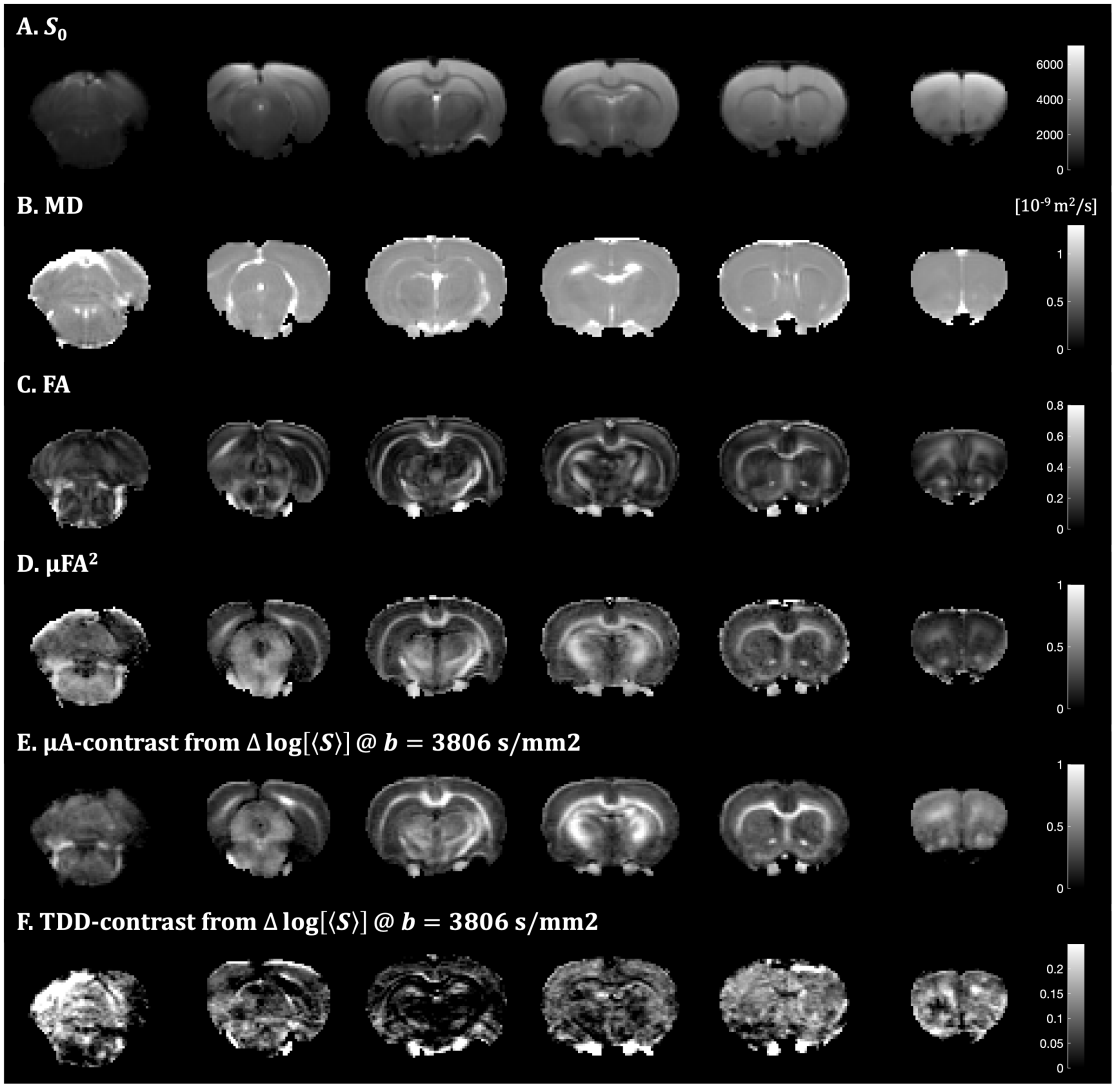
Contrast and parameter maps from the in vivo rat brain measured with STE and SPAS encodings. Panels (A)-(F) correspond to the parameter maps in the same order as inFigure 5. Compared with the fixed tissue (Fig. 5), MD was higher*in vivo*, but*S*_0_, FA, and µFA were within similar ranges as in the fixed tissue. Overall, the µA-based contrast was elevated, and the TDD-based contrast reduced.

**Fig. 10.**
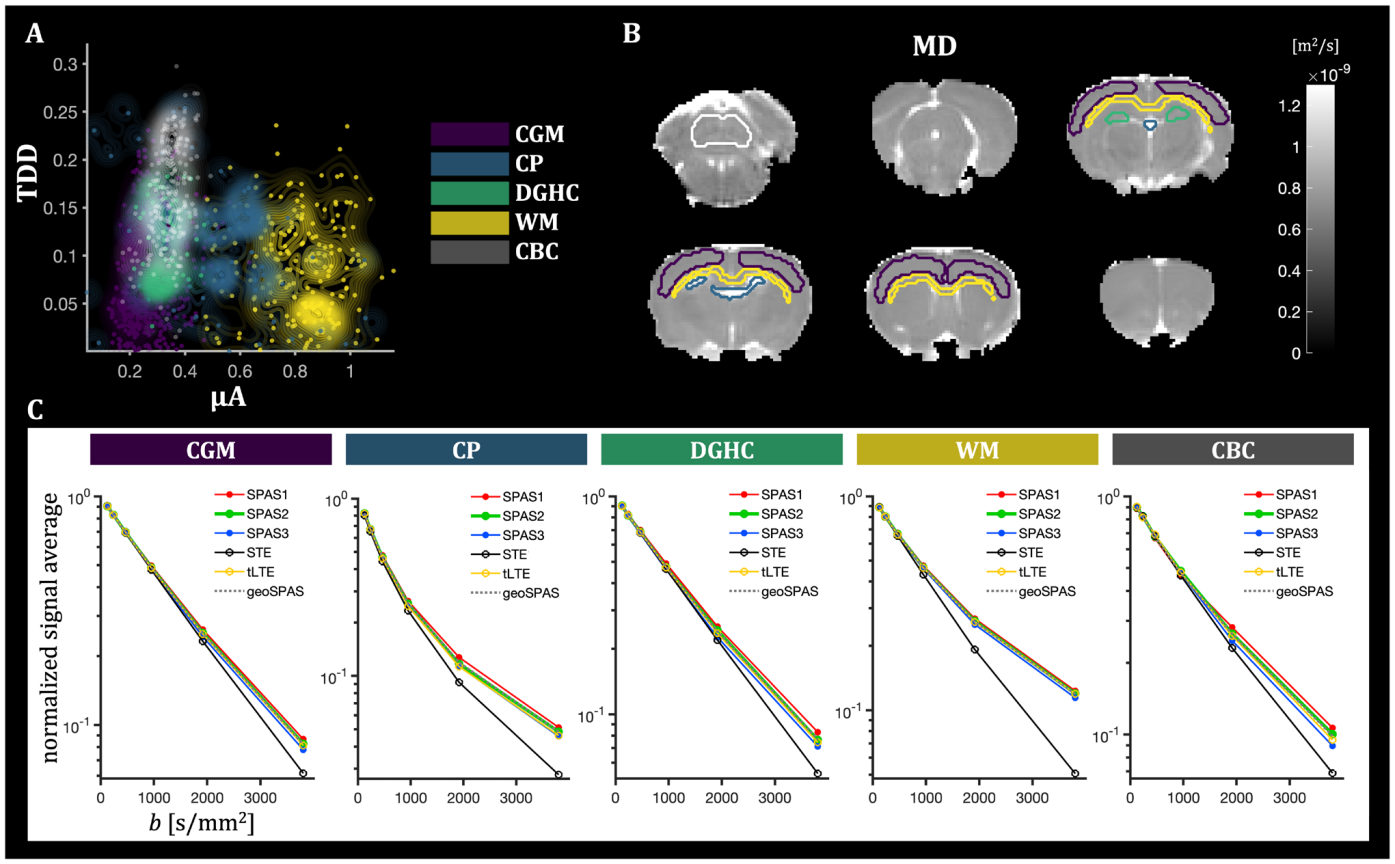
Voxel-wise scatter plot of µA and TDD contrasts (A), ROI selection (B), and ROI-average signals (C) for different regions of the in vivo rat brain. As inFigure 7, the regions outlined on the MD maps include*cortical gray matter*(CGM),*choroid plexus*(CP),*dentate gyrus of the hippocampus*(DGHC), and the*white matter*(WM). The signals from SPAS1–SPAS3 as well as the tLTE and geoSPAS are consistently ordered as expected. Compared with the fixed brain (Fig. 7), the*in vivo*results exhibit similar µA-TDD contrasts for the CGM and WM, but relatively lower µA in the DGHC. The TDD-contrast is generally reduced. Relatively low µA and the highest TDD contrasts were observed in the cerebellar cortex (CBC), which was only visible*in vivo*.

*In vivo*results are shown inFigures 9and10. Compared with the fixed tissue, MD was higher*in vivo*while FA and µFA remained in similar ranges (Fig. 9). Notably, the µA-based contrast was more pronounced*in vivo*(Fig. 9F), whereas the TDD-based contrast was generally lower (Fig. 9E). The relationship between both contrasts is shown in the TDD-µA joint contrast map inSupplementary Figure S6, corresponding to the fixed tissue case (Fig. 6). Similar µA-TDD contrasts were found*in vivo*for the CGM and WM, but relatively lower µA in the DGHC (Fig. 10). Due to the larger coverage of the coil in the*in vivo*setup, the cerebellar cortex (CBC) was visible*in vivo*, where relatively low µA and the highest TDD contrasts were observed. The generally lower TDD contrast*in vivo*is reflected also on the µFA parameter maps and their differences for SPAS LTEs (Supplementary Fig. S7), which were less pronounced compared with the fixed tissue (Fig. 8;Supplementary Fig. S5).

## 5 Discussion

This work considers the effects of TDD in tensor-valued diffusion encoding, addressing the need for tuning gradient waveforms for equal sensitivity to restricted diffusion. The need for a systematic approach to tuning in tensor-valued encoding was first highlighted inLundell, Nilsson, Dyrby, et al. (2019). The theoretical work presented inLundell and Lasič (2020)identified the spectral trace as the “tuning lever” determining MD. The spectral trace allowed estimating MDs from direction-averaged signals for a wide range of encodings with STE and LTE waveforms with varying degrees of motion compensation and restriction encoding, enabling estimation of cell sizes in heart tissues (Lasič et al., 2022). However, previous work did not propose general strategies for tuning different b-tensors or identifying waveform projections with maximally different restriction encoding.

We have proposed two strategies for tuning b-tensors of different shapes and thereby obtain unbiased assessment of µA in the presentence of TDD. By tuning, we refer to the matching of apparent mean diffusivities (seeEq. (15)). One b-tensor is considered as a tuning reference and the other as the tensor being tuned to the reference. We remark that in the presence of restricted diffusion, µA may depend on the encoding timing parameters (Ianuş et al., 2017;Lasič et al., 2020;Lundell et al., 2021;Teh et al., 2021). However, the first order effects of restricted diffusion, as governed by tuning, remain constant between LTE and STE (Lasič et al., 2022). We have demonstrated the case of tuning LTE to STE (Figs. 1and2). Furthermore, we have introduced the concept of spectral anisotropy (SA) and defined the associated spectral principal axis system (SPAS) of encoding tensors. We have demonstrated how the SPAS can be used to generate independent contrast maps due to µA and TDD within a single multidimensional diffusion-encoding protocol.

The first tuning approach selects waveform projections such that the apparent mean diffusivity (MD) matches that of the reference encoding tensor (tLTE inFig. 2). This approach provides a relative tuning tailored to different diffusion spectra. The smooth nature of the diffusion spectrum and smooth variation of encoding power distribution from tensor projections warrant robustness of this approach, reflected by the rather constant peak positions on the tuning landscapes for restricted diffusion in a wide range of sizes (Fig. 3). This robustness holds even for weaker diffusion dispersion, for example, in white matter, where the diffusion spectrum is flatter at low frequencies. In such cases, the tuning contours exhibit less directional variation, but the tuning remains determined by the stable position of the contours rather than their depth. While absolute tuning changes with the diffusion spectrum, the key factor is the relative tuning between the tensors. This is akin to tuning an orchestra, where the absolute pitch may shift, but the instruments remain tuned relative to each other. Importantly, tuning with spherical geometry can be generalized to other shapes, as long as the isotropic diffusion spectrum${\bar{\lambda }}_{\text{iso}}(\omega )$inEq. (15)approximates the diffusion spectrum in spheres of appropriate size. This tuning approach can also be applied to non-STE waveforms, as the tuning is determined by the encoding spectral traces(ω). Furthermore, it is independent of the gradient moments and can, therefore, be applied to waveforms with arbitrary flow compensation properties. The flexibility of this approach allows for a degree of optimization, where some tuning could be sacrificed to better suit hardware constraints (optLTE vs. tLTE inFig. 2). We speculate that this approach of deriving the tLTE could also be tailored for incoherent flow, which is characterized by an exponential velocity autocorrelation function (Kennan et al., 1994) and thus by a Lorentzian diffusion spectrum. Such adaptation could be useful for probing time-dependent anisotropic perfusion.

The second tuning approach aims to match the spectral traces of different encoding tensors precisely, making the tuning independent of the diffusion spectrum. This is achieved through geometric averaging of signals (geoSPAS) from any orthogonal set of encoding projections (Eqs. (15)and(19)). The SPAS provides a unique orthogonal set of LTE projections (SPAS1–SPAS3) with maximally different sensitivities to restricted diffusion, inherent in a spectrally anisotropic reference tensor (STE). In this context, a high SA of the encoding is valuable for distinguishing TDD contrast alongside µA contrast or the µFA map.

The competing effects of µA and TDD contrasts in different restricted geometries are illustrated inFigure 4. Signals decrease from SPAS1 to SPAS3 encodings, while the tuned encodings (tLTE and optLTE) yield intermediate signals approximately matching the geoSPAS signals. At higher b-values (2^nd^cumulant), the difference between STE and geoSPAS reflects µA, while the difference between the SPAS-LTEs reflects TDD. The simulations presented inFigure 4and the previous Monte-Carlo simulations (see supplementary information inLundell, Nilsson, Dyrby, et al., 2019) suggest that SA in STE has a relatively small effect on the second cumulant, while tuning (first cumulant) is crucial for accurate µA assessment. As previously observed, the µA contrast depends on restriction scale relative to encoding time (Ianuş et al., 2017;Lasič et al., 2020;Lundell et al., 2021;Teh et al., 2021). However, it is crucial that LTE is tuned to STE for TDD to not confound the µA assessment (Lasič et al., 2022). Further analysis of SA and restricted diffusion effects in tensor-valued encoding is available in the Supplementary Materials section.

The multidimensional SPAS protocol yielded parameter maps (MD, FA, µFA) as well as µA- and TDD-based contrast maps via signal subtraction for the fixed tissue (Fig. 5) and*in vivo*(Fig. 9). While the parameter maps were generated based on higher-order cumulants from the joint fit of STE and LTE data, the µA- and TDD-based contrast maps were derived in a model-free manner by direct signal subtraction at high b-values. Although these results demonstrate the feasibility of generating µA- and TDD-based contrasts in both ex vivo and in vivo settings, it remains to be determined how reproducible these contrasts are across different samples and experimental conditions. It was crucial that the µFA parameter maps and the µA-based contrast maps use the geoSPAS signals along with the STE signals to ensure tuning in terms of equal MD values and thus reduce the confounding effects of TDD. While the geoSPAS signal only accounts for first order attenuation terms, it overlaps well with the tLTE signals (Figs. 7and10), suggesting that higher order terms are similarly represented by the different LTE waveforms. The µA- and TDD-based contrast maps display two independent contrasts, which can be combined to create color-coded TDD-µA joint contrast maps (Fig. 6;Supplementary Fig. S6), or used as a model-free approach for tissue segmentation (Supplementary Fig. S4). These joint contrast maps provide an integrative view of spatial relationships between cell size (TDD) and anisotropy (µA), facilitating identification of tissue regions with unique microstructural properties. Because the MD values from geoSPAS and STE are equal, the µA-based contrast maps, generated by subtractinglog[〈S〉], are proportional to the differences in diffusion variance (or “mean kurtosis”). In contrast, the TDD-based contrast maps, created by subtracting the signals from SPAS1 and SPAS3 encodings, reflect both MD and diffusion variance differences due to TDD (seeEq. (3)). It should be noted that the µA and TDD maps are generated exclusively from images with the highest b-value. These maps could easily be improved by averaging more high-b data, rather than acquiring additional low-b data. In addition, using only high b-values helps reduce artifacts such as CSF partial volume effects and Gibbs ringing, contributing to cleaner contrast.

The importance of tuning for unbiased µFA maps is illustrated inFigure 8and inSupplementary Figures S5 and S7. Note that just as MD, also µFA is “apparent” and depends on the TDD, but tuning of tensor-valued encoding is crucial for unbiased µFA estimation and separation of the isotropic and anisotropic sources of diffusion variance. Without this consideration, TDD could lead to severely biased results and interpretations particularly in preclinical diffusion-weighted imaging experiments with strong gradients that combine tensor-valued encodings such as STE and LTE with very different sensitivities to restricted diffusion (He et al., 2021). With the SPAS protocol, the “corrupted” µFA maps could be differentiated and thus potentially provide an additional valuable contrast (Fig. 8D, E;Supplementary Figs. S5D–F and S7D–F).

Elevated TDD has been observed with oscillating gradients in the*ex vivo*mouse brain, particularly in the granular layers of the cerebellum and hippocampus (Aggarwal et al., 2012,^2020^). A similar trend has been observed in the cerebellum of the*ex vivo*monkey brain (Lundell et al., 2015;Lundell, Nilsson, Dyrby, et al., 2019). OGSE experiments in mice have shown similar results, with higher MD*ex vivo*and reduced TDD effects*in vivo*, consistent with our observations (Wu et al., 2014). The underlying mechanisms for the*in vivo*versus*ex vivo*differences may be related to neuronal beadings—a morphological feature suggested to drive diffusion changes during hypoxia and stroke (Budde & Frank, 2010). Interestingly, the decreased MD and increased TDD in fixed tissue resemble patterns observed in an*in vivo*stroke model, where TDD changes were recently demonstrated using tensor-valued encoding with differently tuned LTE waveforms (Zhou et al., 2024). This suggests that hypoxia and fixation may induce similar microstructural changes. Additionally, TDD effects have been shown to vary across cortical regions, such as the differences between the soma-rich occipital cortex and frontal regions (Lundell, Nilsson, Dyrby, et al., 2019). In this work, we also observed elevated TDD in specific cellular layers (Fig. 5). The comparison of the choroid plexus signals and simulation with spheroidal restrictions suggest a potentially high density of cuboidal epithelial cells, fibroblasts, or macrophages (Saunders et al., 2023). We speculate that the radial patterns visible within cortical gray matter on the µFA maps (Fig. 5D) could be related to cortical columns with high neurite densities or are associated with blood vessels, as suggested by the radial patterns observed in the cortex on the MD map (Fig. 5B). A bright tangential layer on the TDD map (Fig. 5F) possibly corresponds to the soma-rich layer 4 (Narayanan et al., 2017) with cell radii ~5 µm (Meitzen et al., 2011).

Alternative approaches to probe microscopic anisotropy rely on double diffusion encoding, which is a special case of employing planar tensor encoding (PTE) along with LTE (Jespersen et al., 2013). Tuning between LTE and PTE is in this case ensured by using long mixing times between the two encoding blocks, assuming equal timing is used in the two blocks. With double diffusion encoding, long mixing times could in principle also allow to independently vary tuning and SA for additional specificity to restricted anisotropic diffusion (Lundell et al., 2018). A related approach employing double oscillating gradients can be used to map time-dependent µA (Ianuş et al., 2017,^2018^;Nielsen et al., 2018). However, such approaches rely on specific encoding protocols with limited degrees for optimizations, and importantly, cannot assess the relationship between µA and TDD within a single multidimensional protocol with minimal confounding factors.

Some degree of SA is generally unavoidable in tensor-valued encoding, which uses asynchronous gradient waveforms along orthogonal axes. This needs to be considered in tuning. Due to SA, STE may no longer be rotationally invariant (isotropic encoding), but depend on the relative angle between the encoding and the main diffusion axis (Jespersen et al., 2019;Lasič et al., 2021;Lundell et al., 2018;Lundell, Nilsson, Dyrby, et al., 2019;Lundell & Lasič, 2020). While this effect has not been observed in the human brain*in vivo*with encoding times of about 75 ms (Szczepankiewicz, Lasič, et al., 2019), it has been demonstrated in a fixed monkey brain on a preclinical scanner with encoding times of about 25 ms (Lasič et al., 2021) and in prostate cancer mice models with encoding times of about 15 ms (Szczepankiewicz et al., 2023). When TDD is significant, multiple rotations of the STE are required for the powder-averaged signal to be truly isotropic.

Identifying the SPAS is instrumental for taking SA into consideration. In addition to the low-pass filtering approach for defining the SPAS, we proposed alternative SPAS definitions based on encoding spectral moments (seeSection 2.4andSupplementary Materials). The spectral moments also relate to the generalization of centroid encoding frequencies from LTE (Arbabi et al., 2020) to tensor-valued encoding, which may be useful for gauging SA.

The proposed approach does not aim to maximize sensitivity to TDD per se, but rather exploits the available SA of the reference encoding. While we have here used the SPAS to obtain encodings with maximally different sensitivities to TDD, one could instead minimize such differences by intermediate rotations of the SPAS, thus providing yet another means of tuning, where a single projection could be used instead of employing geometric averaging of signals. Such approach resembles an approximate tuning used previously, where a waveform along a single axis of STE could be used for an approximately tuned LTE (Lundell, Nilsson, Dyrby, et al., 2019). We have since identified the spectral trace as the key property for tuning b-tensors (Lundell et al., 2021), which was instrumental for the herein proposed tuning approaches. Our analysis elucidates the role of SA in the geometric averaging approach to tuning. It also suggests that using reference tensors like STE with axially symmetric SA, resembling the q-MAS trajectory (Topgaard, 2013) used inLundell, Nilsson, Dyrby, et al. (2019), could enable faster acquisitions of “tuned” signals via weighted geometric averaging of only two instead of three SPAS-LTEs.

An important aspect of our tuning approach is the use of b-tensors that are derived from the reference b-tensor, inheriting q-trajectory properties, which can be optimized for different hardware constraints (Sjölund et al., 2015) including gradient moment nulling (Lasič et al., 2020;Szczepankiewicz, Teh, et al., 2021) or minimization of concomitant field effects (Szczepankiewicz, Westin, et al., 2019). While it is tempting to consider additional LTEs designed to boost TDD sensitivity instead of employing derived LTEs, such an approach would require a separate protocol, potentially prolong scan time, and potentially compromise direct comparison with µA, if the average restriction encoding is not matched. We expect that the proposed SPAS-based approach could be adopted for any pair of b-tensors provided that the tensors being tuned are derived from the reference tensors via affine transformations, that is. by scaling the original q-trajectories.

There are some limitations of the proposed approach. The method relies on the spectral-domain analysis in the Gaussian phase approximation, neglecting effects of intra-compartmental kurtosis (Henriques et al., 2020;Jespersen et al., 2019) and exchange (Chakwizira, Westin, et al., 2023;Chakwizira, Zhu, et al., 2023;Lasič et al., 2024;Olesen et al., 2022;Pfeuffer et al., 1998). The accuracy of such approach would need to be tested with Monte-Carlo simulations for different substrates (Chakwizira, Westin, et al., 2023;Lundell, Nilsson, Dyrby, et al., 2019; Lundell, Nilsson,Szczepankiewicz, et al., 2019). Only simple restricted diffusion models for monodispersed compartments with a single size and shape were used in simulations and for deriving tuned projections. Additional simulations could be used to address precision and optimize the protocol in various experimental settings. Biophysical models comprising heterogeneous restricted compartments with exchange (Chakwizira, Westin, et al., 2023;Nilsson et al., 2018), hindered extracellular diffusion spaces (Novikov et al., 2011,^2014^;Novikov, Fieremans, et al., 2018), or time-dependent perfusion (pseudo-diffusion) (Kennan et al., 1994) could be considered in future studies. Depending on the nature of TDD effects, modulating the first order effects, as in our approach, or the higher order terms such as kurtosis could have complementary value. Although the proposed method does not account for exchange, we note that in our experiments, the exchange weighting times (Ning et al., 2018) were relatively short and within a narrow range (Γ = 1.6-1.8 ms), approximately 20 times shorter than those used in studies specifically targeting exchange and TDD (Chakwizira, Westin, et al., 2023;Chakwizira, Zhu, et al., 2023). Since the tuned encoding inherits encoding features from the reference, potentially confounding effects of exchange from different tensor projections could be minimized by striving for reference b-tensors with minimal directional variation in exchange sensitivity.

This work is relevant to several studies of microscopic anisotropy employing tensor-valued encoding, including q-trajectory imaging and diffusional variance decomposition, applied, for example, on brain tumors (Brabec et al., 2022;Li et al., 2021;Nilsson et al., 2018;Szczepankiewicz et al., 2016), various neurological conditions (Afzali et al., 2022;Andersen et al., 2020;Lampinen, Zampeli, et al., 2020;Syed Nasser et al., 2022), and in various organs such as kidney (Nery et al., 2019), prostate (Langbein et al., 2021;Nilsson et al., 2021), breast (Cho et al., 2022), and heart (Teh et al., 2023). Considering b-tensor tuning and TDD is particularly necessary in preclinical settings employing shorter encoding times (He et al., 2021;Rios-Carrillo et al., 2023;Yon et al., 2020). Our findings support the potential of this method for studying microstructural tissue changes in various animal disease models, including conditions such as inflammation, stroke, and tumors. Recently, the ability of tensor-valued encoding to probe TDD in the human brain has been demonstrated (Johnson, Irfanoglu, et al., 2023;Johnson, Ross, et al., 2023), employing centroid encoding frequencies within the range of 6.5–21 Hz, reaching a maximum of only 11 Hz at*b*= 3000 s/mm^2^. The encoding frequencies employed on the human scanner were notably lower than the frequencies in our study, which ranged from 21 to 69 Hz. This observation underscores the feasibility of applying SPAS-based protocols in human studies, not only for mapping microscopic anisotropy but also for detecting TDD. This is particularly relevant in conjunction with the emerging specialized gradient coils, such as MAGNUS (Foo et al., 2020), which offer exciting possibilities for microstructure imaging. The proposed method could help unveil microstructural features and play an important role in refining biophysical models of white matter (Assaf et al., 2004,^2008^;Coelho et al., 2022;Jelescu et al., 2016;Nilsson et al., 2013;Novikov, Fieremans, et al., 2018), gray matter (Afzali et al., 2020;Jelescu et al., 2022;Novikov et al., 2011;Olesen et al., 2022;Palombo et al., 2020), or tumor tissues (X. Jiang et al., 2017;Nilsson et al., 2018;Reynaud, 2017;Reynaud et al., 2016b,2016a).

## 6 Conclusion

In this work, we addressed the confounding effects of time-dependent diffusion (TDD) due to restricted diffusion in tensor-valued encoding and proposed strategies for tuning b-tensors to enable an unbiased assessment of microscopic anisotropy (µA). We introduced two approaches for tuning b-tensors to minimize the confounding effects of restricted diffusion. The first approach involves identifying encoding tensor projections that yield equal mean diffusivities, ensuring reliable tuning across various diffusion spectra for unbiased µA assessments. The second approach utilizes geometric averaging of signals from multiple LTEs, providing more robust tuning that is independent of the underlying diffusion spectra.

Additionally, the same encoding waveforms used in the geometric averaging approach for probing µA can also be used to probe an independent contrast due to TDD. This is facilitated by introducing the concepts of spectral anisotropy (SA) and the spectral principal axis system (SPAS) in tensor-valued encoding. Projections along the SPAS axes generate encodings with maximally different sensitivities to TDD, enabling the creation of two independent contrasts—µA and TDD—within a single multidimensional diffusion encoding protocol.

In the proposed framework, SA is not seen as a limitation that breaks the rotational invariance of STE, but rather as a valuable encoding feature that provides an additional independent contrast. The approach is flexible, accommodating free gradient waveforms that can be optimized to suit various hardware constraints. SPAS encodings provide complementary information to the b-tensor anisotropy, which is necessary to study orientationally dispersed, time-dependent anisotropic diffusion.

A minimal experimental protocol using SPAS encodings at a single b-value could serve as a model-free method to generate contrast maps specific to µA and TDD based on signal differences. The methodology shows potential for studying tissue microstructure changes in various disease models and may enable detection of both µA and TDD in the human brain. These methodological advances could refine biophysical models and enhance our understanding of tissue microstructure.

## Supplementary Material

Supplementary Material

## Data Availability

Code used for data analysis and simulations is available on GitHub athttps://github.com/samo-lasic/Lasic_SPAS_ImagingNeuroscience2025.
