# Supplementary material for: Spectral principal axis system (SPAS) and tuning of tensor-valued encoding for microscopic anisotropy and time-dependent diffusion in the rat brain
Source: Imaging Neurosci (Camb). 2025 Jun 11;3:IMAG.a.35. doi: 10.1162/IMAG.a.35 (PMC12319979; doi:10.1162/IMAG.a.35)
Supplement: Supplementary Material [file imag.a.35_supp.pdf]

Supplementary materials

Spectral Principal Axis System (SPAS) and tuning of tensor-valued encoding for microscopic anisotropy and time-dependent diffusion in the rat brain

Samo Lasič[1,2], Nathalie Just[1], Markus Nilsson[3], Filip Szczepankiewicz[4], Matthew Budde[5], and Henrik Lundell[1,6]

1. Danish Research Centre for Magnetic Resonance, Department of Radiology and Nuclear Medicine, Copenhagen University Hospital - Amager and Hvidovre, Copenhagen, Denmark
2. Department of Diagnostic Radiology, Lund University, Lund, Sweden
3. Department of Clinical Sciences Lund, Radiology, Lund University, Lund, Sweden
4. Medical Radiation Physics, Clinical Sciences Lund, Lund University, Lund, Sweden
5. Department of Neurosurgery, Medical College of Wisconsin, Milwaukee, WI, United States
6. Magnetic Resonance Section, DTU Health Tech, Technical University of Denmark, Kgs. Lyngby, Denmark

Corresponding author:
Samo Lasič
Danish Research Centre for Magnetic Resonance, Department of Radiology and Nuclear Medicine, Copenhagen University Hospital - Amager and Hvidovre, Copenhagen, Denmark
Email address: samol@drcmr.dk

1 Impact of time-dependent diffusion on the second cumulant of direction average signal

Effects of time-dependent diffusion (TDD) in tensor-valued encoding have been evaluated using frequency-domain analysis under Gaussian phase approximation (GPA) (Stepišnik, 1993, 1999), ignoring effects of exchange and assuming that the normalized signal attenuation from each compartment is mono-exponential, $E = e^{-\beta}$ (Lundell & Lasič, 2020). Let us summarize the key results focusing on the second cumulant of direction average signal, generalizing the notions defined by Topgaard (Topgaard, 2017). The key question is how restricted diffusion affects the isotropic and anisotropic contributions to the second cumulant.

1.1 Average signal attenuation and apparent diffusivities

After averaging over compartments and encoding directions, the normalized signal is given by the cumulant expansion as

$$\log[\langle E \rangle] \approx -\langle \beta \rangle + \frac{1}{2}[\langle \beta^2 \rangle - \langle \beta \rangle^2]. \quad \text{(S1)}$$

The second cumulant can be expressed as a sum of isotropic and anisotropic apparent diffusion variances (Szczepankiewicz et al., 2019),

$$\frac{1}{2}[\langle \beta^2 \rangle - \langle \beta \rangle^2] = \frac{b^2}{2}[V_\mathrm{I} + V_\mathrm{A}]. \quad \text{(S2)}$$

Based on Eqs. (13)–(14) for a single compartment, we define the apparent diffusivity as

$$\Lambda_{ijk} \equiv \frac{1}{b}\frac{1}{2\pi}\int_{-\infty}^{\infty} s_{ij}(\omega)\,\lambda_k(\omega)\mathrm{d}\omega, \quad \text{(S3)}$$

which depends on the diffusion eigen-spectra $\lambda_k(\omega)$ and the encoding power spectra $s_{ij}(\omega)$. Averaging over all rotations yields

$$V_\mathrm{I} = \frac{1}{9}\sum_{i,j,k,l}\left[\langle \Lambda_{iik}\Lambda_{jjl} \rangle - \langle \Lambda_{iik} \rangle \langle \Lambda_{jjl} \rangle\right] \quad \text{(S4)}$$

and

$$V_\mathrm{A} = \frac{1}{45}\sum_{i,j,k,l}(3\delta_{kl} - 1)\left[3\langle \Lambda_{ijk}\Lambda_{ijl} \rangle - \langle \Lambda_{iik} \rangle \langle \Lambda_{jjl} \rangle\right]. \quad \text{(S5)}$$

Eqs. (S3)–(S5) are the key result of this analysis.

It is important to note that all the summation terms in Eq. (S4) for V_I depends on the diagonal encoding power spectra $s_{ii}(\omega)$ via the apparent diffusivities like Λ_{iik}. Eq. (S4) can thus be rewritten by averaging first and summing over all frequency pairs last,

$$V_{\mathrm{I}} = \left(\frac{1}{2\pi b}\right)^2 \int_{-\infty}^{\infty}\int_{-\infty}^{\infty} s(\omega_1)s(\omega_2)\, V_{\mathrm{iso}}(\omega_1,\omega_2)\; d\omega_1 d\omega_2, \tag{S6}$$

where

$$V_{\mathrm{iso}}(\omega_1,\omega_2) = \langle \lambda_{\mathrm{iso}}(\omega_1)\lambda_{\mathrm{iso}}(\omega_2)\rangle - \langle \lambda_{\mathrm{iso}}(\omega_1)\rangle\langle \lambda_{\mathrm{iso}}(\omega_2)\rangle, \tag{S7}$$

$s(\omega)$ is the spectral trace (see Eq. (11)) and $\lambda_{\mathrm{iso}}(\omega)$ are isotropic diffusion spectra (c.f. Eq. (16)). Eq. (S6) tells us that the isotropic variance V_{I} only depends on the spectral trace of b-tensors. In case of the anisotropic variance V_{A}, the change of summation order is not possible because of the mixed summation terms in Eq. (S5), containing diagonal and off-diagonal spectral components via the terms like Λ_{iik} and Λ_{ijk}, respectively. We will return to this important point later. As we will see, the anisotropic variance V_{A} is sensitive to b-tensor anisotropy and, crucially for restricted diffusion, V_{A} depends also on the directional dependence of the encoding power distribution (*spectral anisotropy*).

1.2 Anisotropy of b-tensors for probing anisotropic Gaussian diffusion

For Gaussian diffusion,

$$\Lambda_{ijk} \equiv \frac{b_{ij}}{b}\lambda_k \tag{S8}$$

$$V_{\mathrm{I}} = \frac{1}{9}\sum_{i,j}\left[\langle \lambda_i\lambda_j\rangle - \langle\lambda_i\rangle\langle\lambda_j\rangle\right] = \langle D_{\mathrm{iso}}^2\rangle - \langle D_{\mathrm{iso}}\rangle^2 \tag{S9}$$

and

$$V_{\mathrm{A}} = \tfrac{2}{5}\zeta_{\mathrm{b}} V_{\lambda}, \tag{S10}$$

where

$$\zeta_{\mathrm{b}} = \frac{1}{2b^2}\sum_{i,j}(3b_{ij}^2 - b_{ii}b_{jj}) \tag{S11}$$

is the *shape factor* $0 \le \zeta_{\mathrm{b}} \le 1$ (Topgaard, 2017) and

$$V_{\lambda} = \frac{1}{9}\sum_{i,j}(3\delta_{ij} - 1)\langle\lambda_i\lambda_j\rangle \tag{S12}$$

is the average variance of diffusion eigenvalues or more simply the average diffusion anisotropy (Topgaard, 2017).

1.3 Spectral anisotropy of b-tensors: low-frequency approximation

While the *shape factor* quantifies b-tensor anisotropy, quantification of *spectral anisotropy* is more difficult due to the complex interaction between encoding and diffusion at different frequencies (Eq. (S5)). However, some intuition of the effects of *spectral anisotropy* can be gained based on the low-frequency approximation of the restricted diffusion spectrum (Lasič et al., 2009), which has been previously used in the

context of encoding along a single direction (Burcaw et al., 2015; Lasič et al., 2006; Nilsson et al., 2017; Stepišnik, 1993; Stepišnik et al., 2006). The diffusion spectrum can be written as

$$D_{ij}(\omega) \approx D_{ij} + R_{ij}\,\omega^2, \tag{S13}$$

where D_{ij} is the long-time (Gaussian) diffusion tensor related to the tortuosity constant (Lasič et al., 2009; Stepišnik et al., 2006) and R_{ij} is the effective *size sensitivity tensor*, reflecting pore shape as the low-frequency approximation of the diffusion eigen-spectra (Lundell & Lasič, 2020). Note that the sample may consist of a mixture of Gaussian and restricted diffusion compartments.

For simple geometries, the eigenvalues of R_{ij} are given by

$$\rho_i = \frac{r_i^4}{D_0}\Gamma_i, \tag{S14}$$

where r_i is the compartment size, D_0 is the short-time diffusivity, and Γ_i is the geometric factor (Nilsson et al., 2017).

Inserting Eq. (S13) into Eq. 13 yields

$$\beta \approx \sum_{i,j}\left[b_{ij}\,D_{ij} + m_{ij}\,R_{ij}\right], \tag{S15}$$

where

$$m_{ij} = \frac{1}{2\pi}\int_{-\infty}^{\infty} s_{ij}(\omega)\,\omega^2\,\mathrm{d}\omega \tag{S16}$$

is the *encoding spectral variance tensor*. This tensor is representative of *spectral anisotropy*. For example, while STE features an isotropic b-tensor, the corresponding m-tensor is generally anisotropic, which is related to the color-coding in **Figure 1** and the spectral principal axes can be determined from the eigenvectors of the m-tensor (**Figure S1**).

The separation of the attenuation factor into the Gaussian and restricted parts provides more tractable solutions for the cumulants and allows drawing analogies from the Gaussian diffusion case. The first cumulant (c.f. Eq. 15) is given by

$$\langle\beta\rangle \approx b\,\overline{D}_{\mathrm{iso}} + m\,R_{\mathrm{iso}}, \tag{S17}$$

where

$$m = \sum_{i=1}^{3} m_{ii} \tag{S18}$$

is the trace of the m-tensor and

$$R_{\text{iso}} = \frac{1}{3}\sum_{i=1}^{3} R_{ii}$$

represents isotropic size sensitivity.

The variances contributing to the second cumulant in Eq. (S2) are given by

$$b^2\, V_{\text{I}} = b^2\, V_{\text{DI}} + m^2\, V_{\text{RI}} + 2b\, m\, C_{\text{DRI}} \tag{S19}$$

and

$$b^2\, V_{\text{A}} = \frac{2}{5}\left[b^2\, \zeta_{\text{b}}\, V_{\lambda} + m^2\, \zeta_{\text{m}}\, V_{\rho} + 2\, b\, m\, \zeta_{\text{bm}}\, C_{\lambda\rho}\right]. \tag{S20}$$

The isotropic variance V_{I} is composed of the contribution from the Gaussian diffusion tensors, V_{DI}, corresponding to the definition in Eq. (S9), the isotropic variance of size sensitivity tensor, given by

$$V_{\text{RI}} = \frac{1}{9}\sum_{i,j}\left[\langle\rho_i\rho_j\rangle - \langle\rho_i\rangle\langle\rho_j\rangle\right] = \langle R_{\text{iso}}^2\rangle - \langle R_{\text{iso}}\rangle^2 \tag{S21}$$

and the covariance

$$C_{\text{DRI}} = \frac{1}{9}\sum_{i,j}\left[\langle\lambda_i\rho_j\rangle - \langle\lambda_i\rangle\langle\rho_j\rangle\right] = \langle D_{\text{iso}}R_{\text{iso}}\rangle - \langle D_{\text{iso}}\rangle\langle R_{\text{iso}}\rangle. \tag{S22}$$

The anisotropic variance V_{A} is composed of the of the Gaussian contribution V_{λ} from Eq. (S12), the average anisotropy of size sensitivity tensors

$$V_{\rho} = \frac{1}{9}\sum_{i,j}(3\delta_{ij} - 1)\langle\rho_i\rho_j\rangle \tag{S23}$$

and the covariance

$$C_{\lambda\rho} = \tfrac{1}{9}\textstyle\sum_{i,j}(3\delta_{ij} - 1)\langle\lambda_i\rho_j\rangle. \tag{S24}$$

The shape factor ζ_{b} is defined by Eq. (S11), the shape factor related to the second moment of the encoding spectrum (m-tensor) is

$$\zeta_{\text{m}} = \frac{1}{2m^2}\sum_{i,j}(3m_{ij}^2 - m_{ii}m_{jj}) \tag{S25}$$

and the coupled shape factor is

$$\zeta_{\mathrm{bm}} = \frac{1}{2bm} \sum_{i,j} (3b_{ij} m_{ij} - b_{ii} m_{jj}). \tag{S26}$$

We note that the coupled shape factor ζ_{bm} used in Eq. (S20) was erroneously omitted in Eq. 2.164 in (Lundell & Lasič, 2020). This term reflects the coupling between the anisotropies of the Gaussian and restricted diffusion. This coupling extends to all frequency pairs in the more general case of Eq. (S5), preventing to change the summation order over frequencies and ensembles, as discussed earlier. While the low-frequency approximation may not be accurate, it provides useful intuition to understand the intricate coupling of restricted diffusion effects in tensor-valued encoding.

2 SPAS based on encoding spectral moments

The eigenvectors of the encoding spectral moment tensor, like the spectral variance tensor, could provide an alternative definition of the spectral principal axes system (SPAS). In contrast to the low-pass filtered encoding spectrum, this version of the SPAS (v_1-v_3 in **Figure S1**) involves high-pass filtering.

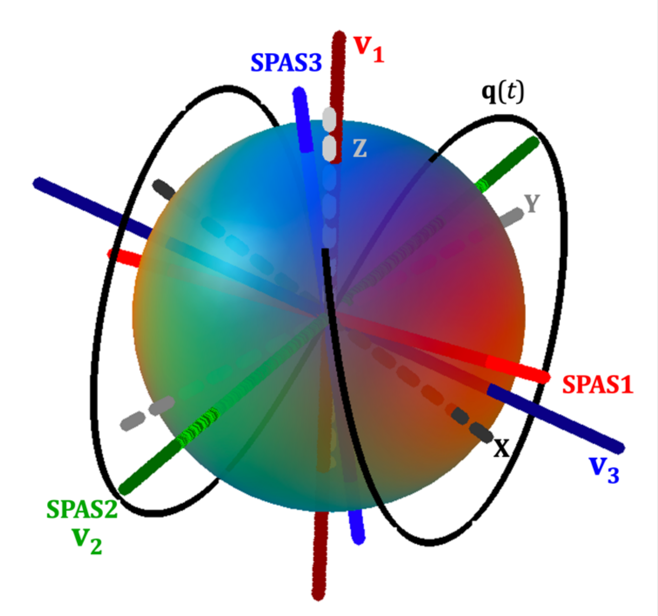

Figure S1: Alternative SPAS based on spectral moments of the spherical tensor encoding (STE). Besides the SPAS featured also in **Figure 1**, the v_1, v_2, v_3 axes correspond to the high-pass filtered STE based on the encoding spectral variance tensor, i.e. the 2nd spectral moment tensor according to Eq. (21) or Eq. (S16). The complementarity of the two SPAS is reflected by the color coding, where power decreases from red to blue for low frequencies in the case of SPAS1-SPAS3 and for high frequencies in the case of v_1-v_3.

3 Experimental results

3.1 Phantom

Reference experiments were conducted on water, confirming that the signals from all encodings overlaps at equal b values. Direction average signals are shown in **Figure S2** and signals for all 12 directions are shown in the **Figure S3**.

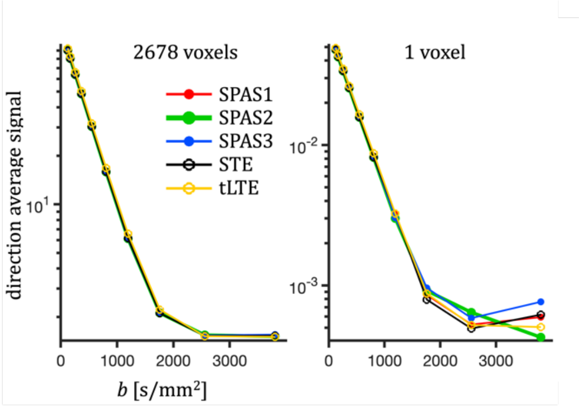

Figure S2: Direction average signals from the water reference using the ex vivo protocol. Shown is the signal average from 2678 voxels (left) and from a single representative voxel (right).

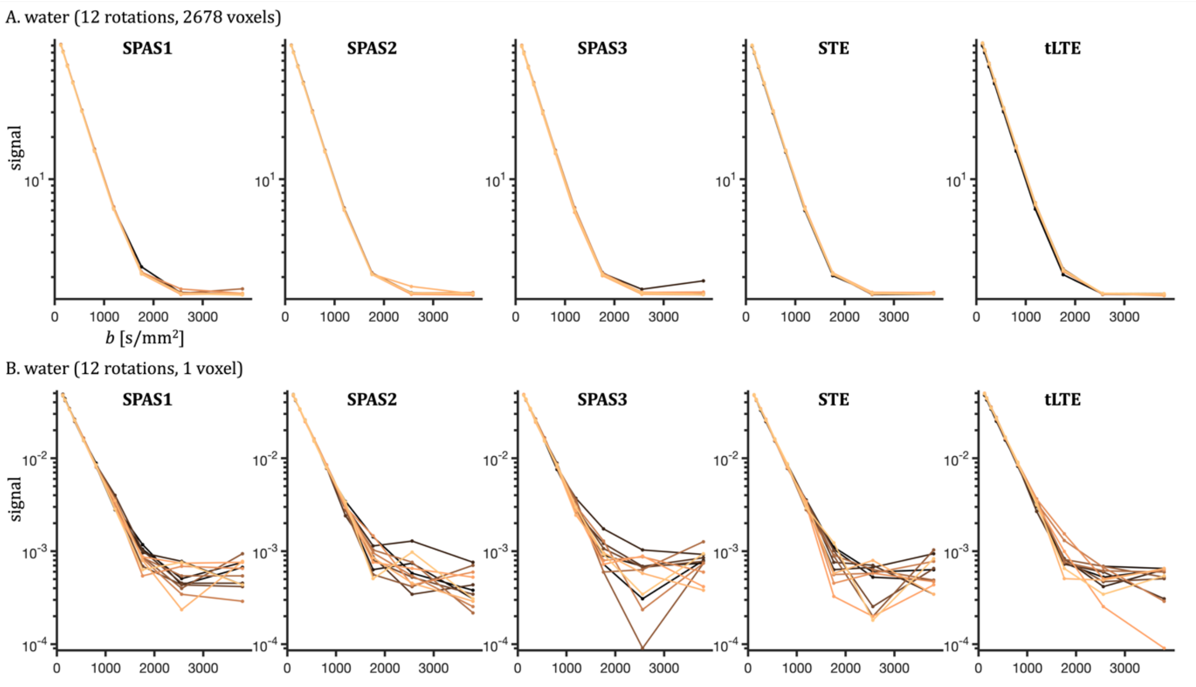

Figure S3: Reference signals from water using the ex vivo protocol. For each diffusion encoding waveform (columns) the signals are shown for all 12 rotations (color coded) as an average from 2678 voxels (A) and for a single voxel (B).

3.2 Fixed rat: Segmentation based on microscopic anisotropy (µA) and time-dependent diffusion (TDD)

Tissue regions with low µA and high TDD contrasts and regions with high µA and low TDD in the fixed brain are outlined in **Figure S4**. This could provide a model-free approach for automatic tissue segmentation.

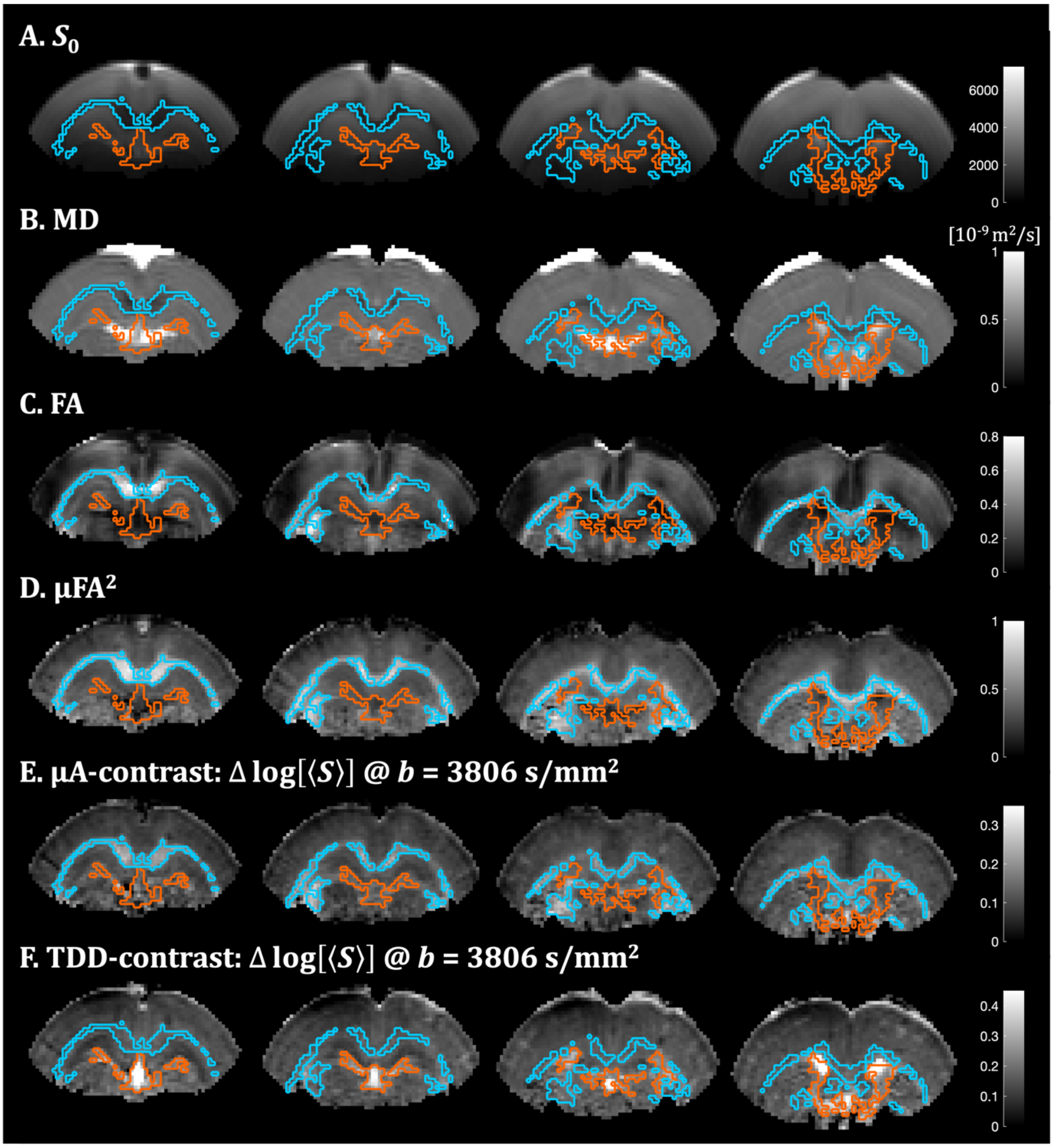

Figure S4: Outlined regions with pronounced µA and TDD contrasts overlayed on the contrast and parameter maps from the fixed rat brain (see Figure 5). Regions with low µA and high TDD contrasts are outlined in orange and regions with high µA and low TDD are outlined in blue.

3.3 Fixed rat: µFA bias

Biased µFA maps from using STE in combination with different non-tuned LTEs (SPAS1, SPAS2 and SPAS3) for the fixed brain are shown in **Figure S5**. Difference between the

"corrupted" μFA maps could potentially provide an additional valuable contrast (see **Figure S5**D-F).

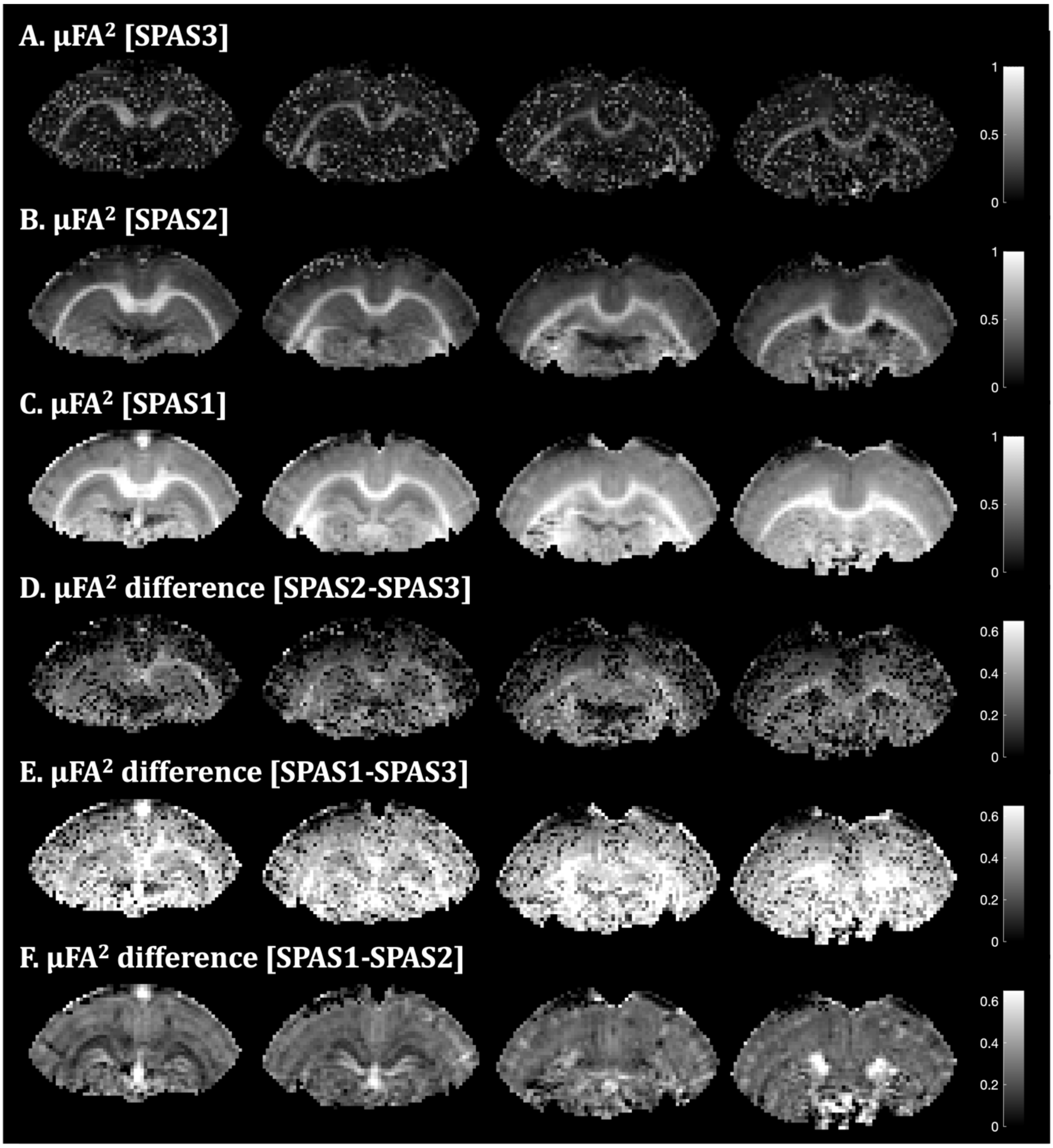

Figure S5: μFA parameter maps for the fixed rat brain estimated from STE and different SPAS LTEs (SPAS1-SPAS3). The μFA maps are biased due to TDD and not tuned SPAS LTEs (A-C). The reduced signal difference between STE and SPAS3 results in reduced μFA and reduced precision (A). The μFA increases for SPAS2 and SPAS1 due to increased signal differences relative to the STE signal (B,C). The μFA difference maps reflect the effects of TDD (D-F). The largest differences are found for SPAS1 and SPAS3 (E) encodings with most different sensitivities to TDD. The fitting noise propagates from μFA with SPAS3 (A) to the differences in D and E. The μFA difference from SPAS1 and SPAS2 (F) is more accurate, and this map is similar to the TDD-based contrast maps in **Figure 5F** obtained by subtracting logarithms of direction averaged signals. However, additional contrast is visible on the μFA difference in panel F (slice 2) between the dentate gyrus of the hippocampus and white matter.

3.4 In vivo rat: Relationship between TDD and µA

The relationship between µA- and TDD-based contrasts *in vivo* are shown as a single color map (mix of red and blue) in **Figure S6**.

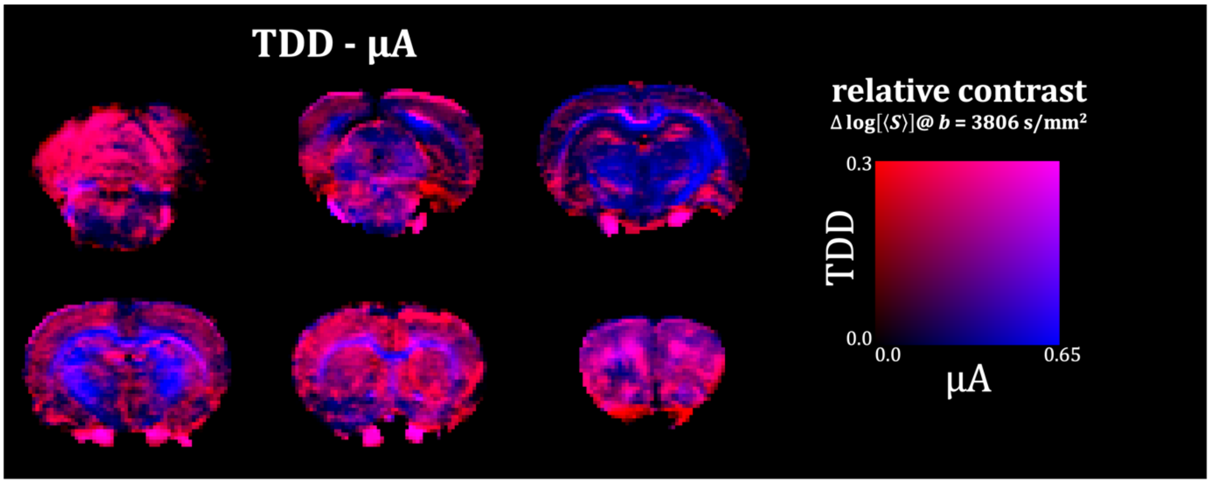

Figure S6: TDD-µA joint contrast map from in vivo rat brain. The separate contrasts due to µA and TDD, shown in **Figure 9E-F**, are here represented as a single color-coded map, where blue encodes the µA and red the TDD contrast. The relative contrasts were limited to 65% and 30% of the µA and TDD contrast ranges, respectively.

3.5 In vivo rat: µFA bias

Biased µFA maps from using STE in combination with different non-tuned LTEs (SPAS1, SPAS2 and SPAS3) *in vivo* are shown in **Figure S7**. Difference between the “corrupted” µFA maps could potentially provide an additional valuable contrast (see **Figure S7**D-F).

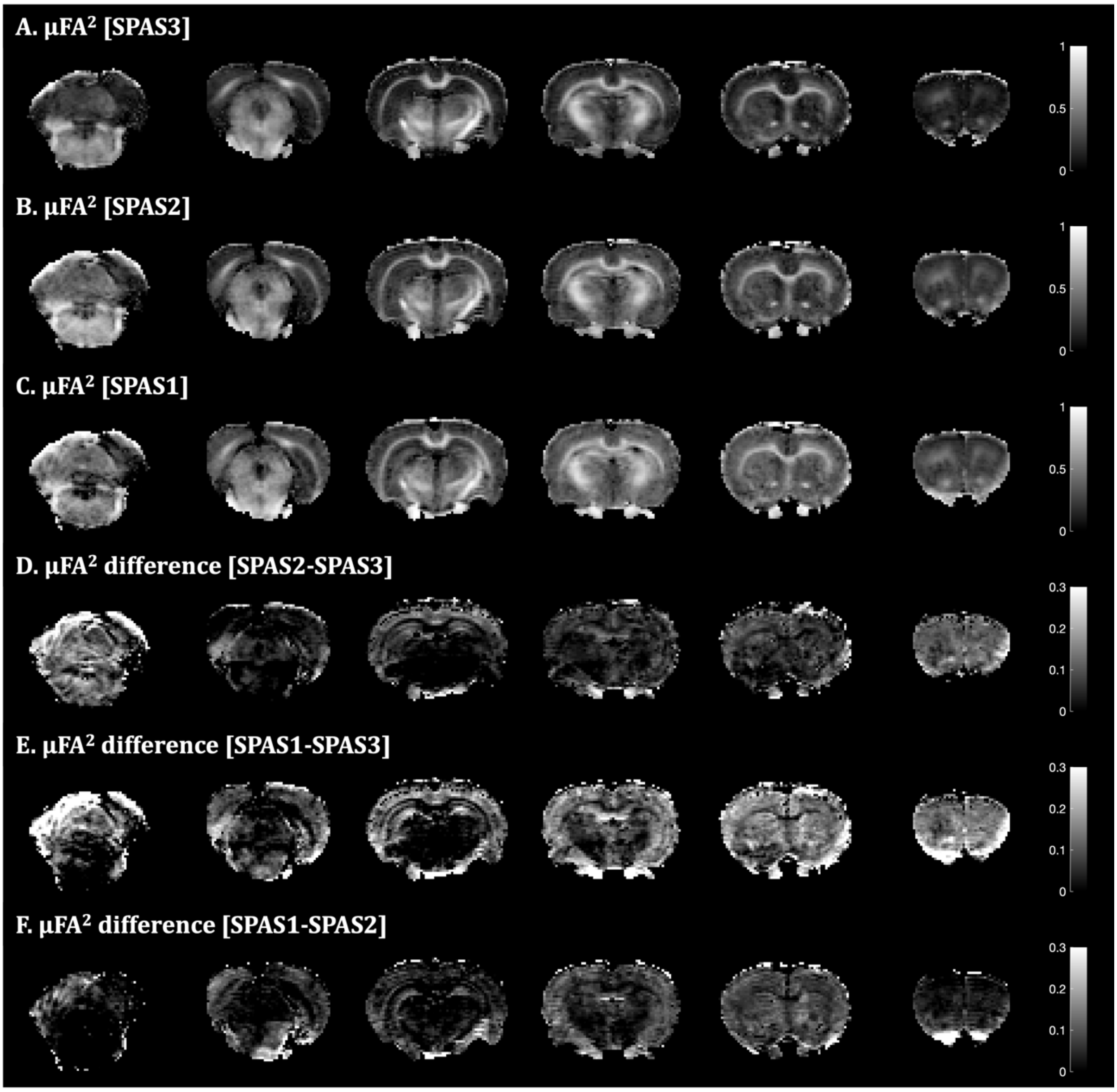

Figure S7: µFA parameter maps for in the vivo rat brain estimated from STE and different SPAS LTEs (SPAS1-SPAS3). The µFA maps are biased due to TDD and not tuned SPAS LTEs (A-C). The µFA difference maps reflect the effects of TDD (D-F). As for the fixed tissue (**Figure S5**), µFA increased from SPAS3 to SPAS1 to to TDD(A-C), but *in vivo* the TDD effect was reduced compared to the fixed tissue. The µFA estimation was more accurate in this case due to sufficient differences between the STE and LTE signals. As expected, the largest differences are found for SPAS1 and SPAS3 (E) encodings with most different sensitivities to TDD. The µFA difference from SPAS1 and SPAS2 (F) is similar to the TDD-based contrast maps in **Figure 9F**. As for the fixed tissue, additional contrast is visible on the µFA difference in panel F (slice 3) between the dentate gyrus of the hippocampus and white matter.

References

Burcaw, L. M., Fieremans, E., & Novikov, D. S. (2015). Mesoscopic structure of neuronal tracts from time-dependent diffusion. *Neuroimage*, *114*, 18–37. https://doi.org/10.1016/j.neuroimage.2015.03.061

Lasič, S., Åslund, I., & Topgaard, D. (2009). Spectral characterization of diffusion with chemical shift resolution: highly concentrated water-in-oil emulsion. *Journal of Magnetic Resonance*, *199*(2), 166–172. https://doi.org/10.1016/j.jmr.2009.04.014

Lasič, S., Stepišnik, J., & Mohorič, A. (2006). Displacement power spectrum measurement by CPMG in constant gradient. *Journal of Magnetic Resonance*, *182*(2), 208–214. https://doi.org/10.1016/j.jmr.2006.06.030

Lundell, H., & Lasič, S. (2020). Diffusion Encoding with General Gradient Waveforms. In D. Topgaard (Ed.), *Advanced Diffusion Encoding Methods in MRI: New Developments in NMR Volume 24* (pp. 12–67). Royal Society of Chemistry. https://doi.org/10.1039/9781788019910-00012

Nilsson, M., Lasič, S., Drobnjak, I., Topgaard, D., & Westin, C. F. (2017). Resolution limit of cylinder diameter estimation by diffusion MRI: The impact of gradient waveform and orientation dispersion. *NMR in Biomedicine*, *30*(7), 1–13. https://doi.org/10.1002/nbm.3711

Stepišnik, J. (1993). Time-dependent self-diffusion by NMR spin-echo. *Physica B*, *183*, 343–350. https://doi.org/10.1016/0921-4526(93)90124-O

Stepišnik, J. (1999). Validity limits of Gaussian approximation in cumulant expansion for diffusion attenuation of spin echo. *Physica B*, *270*, 110–117.

Stepišnik, J., Lasič, S., Mohorič, A., Serša, I., & Sepe, A. (2006). Spectral characterization of diffusion in porous media by the modulated gradient spin echo with CPMG sequence. *Journal of Magnetic Resonance*, *182*(2), 195–199. http://www.ncbi.nlm.nih.gov/pubmed/16844392

Szczepankiewicz, F., Sjölund, J., Ståhlberg, F., Lätt, J., & Nilsson, M. (2019). Tensor-valued diffusion encoding for diffusional variance decomposition (DIVIDE): Technical feasibility in clinical MRI systems. *PLoS ONE*, *14*(3), 1–20. https://doi.org/10.1371/journal.pone.0214238

Topgaard, D. (2017). NMR methods for studying microscopic diffusion anisotropy. In R. Valiullin (Ed.), *Diffusion NMR of confined systems: fluid transport in porous solids and heterogeneous materials, New Developments in NMR no. 9*. Royal Society of Chemistry.
